# The PIDDosome activates p53 in response to supernumerary centrosomes

**DOI:** 10.1101/gad.289728.116

**Published:** 2017-01-01

**Authors:** Luca L. Fava, Fabian Schuler, Valentina Sladky, Manuel D. Haschka, Claudia Soratroi, Lisa Eiterer, Egon Demetz, Guenter Weiss, Stephan Geley, Erich A. Nigg, Andreas Villunger

**Affiliations:** 1 Division of Developmental Immunology, Biocenter, Medical University of Innsbruck, 6020 Innsbruck, Austria;; 2 Department of Internal Medicine VI, Infectious Diseases, Immunology, Rheumatology, Pneumology, Medical University of Innsbruck, 6020 Innsbruck, Austria;; 3 Division of Molecular Pathophysiology, Biocenter, Medical University of Innsbruck, 6020 Innsbruck, Austria;; 4 Biozentrum, University of Basel, 4056 Basel, Switzerland;; 5 Tyrolean Cancer Research Institute, 6020 Innsbruck, Austria

**Keywords:** cell division, centrosome, cytokinesis failure, p53

## Abstract

Centrosomes, the main microtubule-organizing centers in animal cells, are replicated exactly once during the cell division cycle to form the poles of the mitotic spindle. Supernumerary centrosomes can lead to aberrant cell division and have been causally linked to chromosomal instability and cancer. Here, we report that an increase in the number of mature centrosomes, generated by disrupting cytokinesis or forcing centrosome overduplication, triggers the activation of the PIDDosome multiprotein complex, leading to Caspase-2-mediated MDM2 cleavage, p53 stabilization, and p21-dependent cell cycle arrest. This pathway also restrains the extent of developmentally scheduled polyploidization by regulating p53 levels in hepatocytes during liver organogenesis. Taken together, the PIDDosome acts as a first barrier, engaging p53 to halt the proliferation of cells carrying more than one mature centrosome to maintain genome integrity.

## Abstract (precis)

In this study, Fava et al. show that an increase in the number of mature centrosomes (the main microtubule-organizing centers in animal cells), generated by disrupting cytokinesis or forcing centrosome overduplication, triggers the activation of the PIDDosome multiprotein complex, leading to Caspase-2-mediated MDM2 cleavage, p53 stabilization, and p21-dependent cell cycle arrest.

Chromosome segregation errors pose a substantial danger to not only individual cells but also multicellular organisms as a whole. Hence, different control mechanisms secure faithful completion of somatic cell division to maintain genome integrity and tissue homeostasis (Holland and Cleveland 2009). A failure in cell division (cytokinesis) leads to the duplication of the usually diploid chromosomal complement, known as tetraploidization. While genetically programmed cytokinesis abrogation can be an integral part of the development of certain mammalian cell types such as hepatocytes or cardiomyocytes during organogenesis (Pandit et al. 2013), unscheduled cytokinesis failure has been shown to be oncogenic in animal models (Fujiwara et al. 2005). The latter findings go well with the high frequency of whole-genome duplication (WGD) events observed in the course of carcinogenesis in humans. Despite the fact that WGD may limit tumorigenesis under some circumstances (Shoshani et al. 2012), nearly 40% of all human cancers are believed to have undergone at least one WGD event during their evolution (Zack et al. 2013; de Bruin et al. 2014; Dewhurst et al. 2014). Together, this highlights the need to limit the frequency of tetraploidization to prevent malignant disease.

Cytokinesis failure leads to not only WGD but also the acquisition of extra centrosomes, the main microtubule-organizing centers in animal cells (Conduit et al. 2015). While extra centrosomes can be causative of chromosomal instability (CIN) by promoting merotelic kinetochore–microtubule attachments in mitosis (Ganem et al. 2009; Silkworth et al. 2009), they may promote oncogenesis in additional ways unrelated to CIN; e.g., by increasing Rac1 activity that facilitates invasive cellular behavior (Godinho et al. 2014). Conceivably, the induction of centrosome amplification in mouse models can suffice to accelerate cancer onset in the absence of p53 (Coelho et al. 2015; Serçin et al. 2016), but evidence for the lack of such an effect also exists (Kulukian et al. 2015; Vitre et al. 2015). This apparent controversy suggests that the oncogenic potential of extra centrosomes may vary depending on context or cell type, but all studies agree that tissues retaining functional p53 are nonpermissive to propagate supernumerary centrosomes (Coelho et al. 2015; Kulukian et al. 2015; Vitre et al. 2015; Serçin et al. 2016). Perturbations of the centrosome duplication cycle lead to p53 stabilization and p53-dependent cell cycle arrest (Holland et al. 2012; Lambrus et al. 2015; Wong et al. 2015). Of note, while centrosome depletion (or increased mitotic duration) activates p53 via 53BP1 and USP28 (Fong et al. 2016; Lambrus et al. 2016; Meitinger et al. 2016), the molecular machinery activating p53 upon cytokinesis failure or forced generation of extra centrosomes is not understood.

Caspase-2 belongs to a family of endopeptidases that are activated in response to various stressors and are best known for controlling different cell death modalities, including apoptosis, pyroptosis, or necroptosis, thereby limiting or promoting inflammation (Martin et al. 2012; Green et al. 2014). Based on its structure, Caspase-2 classifies as an initiator caspase suggesting an apical position in signaling. Members of this class, such as Caspase-9, Caspase-8, and Caspase-1, are usually activated in high-molecular-weight signaling platforms, such as the apoptosome, the death-inducing signaling complex (DISC), or the inflammasome, and each of these assemble in response to defined cues. These include mitochondrial cytochrome c release from mitochondria, engagement of certain tumor necrosis factor receptor (TNFR) superfamily members by their cognate ligands (e.g., FAS/FASL and TNFR1/TNF), pathogen-associated molecular patterns (PAMPs), or even more universal danger-associated molecular patterns (DAMPs) released from stressed, infected, or dying cells (Martin et al. 2012; Green et al. 2014).

An activating platform containing Caspase-2, PIDD1, and RAIDD (dubbed the PIDDosome) was first described in 2004 (Tinel and Tschopp 2004) and was proposed to induce Caspase-2 activation in response to DNA damage. However, PIDDosome loss-of-function analysis in mice failed to reveal any dysfunction in the DNA damage response (DDR), subsequent apoptosis, or tumor formation upon DNA damage (Manzl et al. 2009, 2012, 2013; Fava et al. 2012). As such, the specific cue activating the PIDDosome remained undefined and led to numerous hypotheses about its function (Bock et al. 2012; Fava et al. 2012).

Here, we set out to identify a genuine trigger for PIDDosome activation and focused on “mitotic catastrophe,” a heterogeneous set of tumor-suppressive cellular responses, including ill-defined cell death modalities as well as senescence, restraining the proliferative capacity of cells experiencing mitotic errors (Vitale et al. 2011). Notably, Caspase-2 has been repeatedly implicated in “mitotic catastrophe,” yet its mode of activation and relevance therein remained disputed (Castedo et al. 2004; Sidi et al. 2008; Fava et al. 2012; Manzl et al. 2013). Our analyses demonstrate that Caspase-2 becomes selectively activated upon cytokinesis failure in a PIDDosome-dependent manner, resulting in MDM2 cleavage, p53 activation, and p21-dependent cell cycle arrest. The PIDDosome responds to the presence of extra mother centrioles, thereby identifying a novel p53-activating pathway directly responding to the presence of extra mature centrosomes.

## Results

### The PIDDosome activates Caspase-2 in response to cytokinesis failure to promote cell cycle arrest

**Figure 1.**
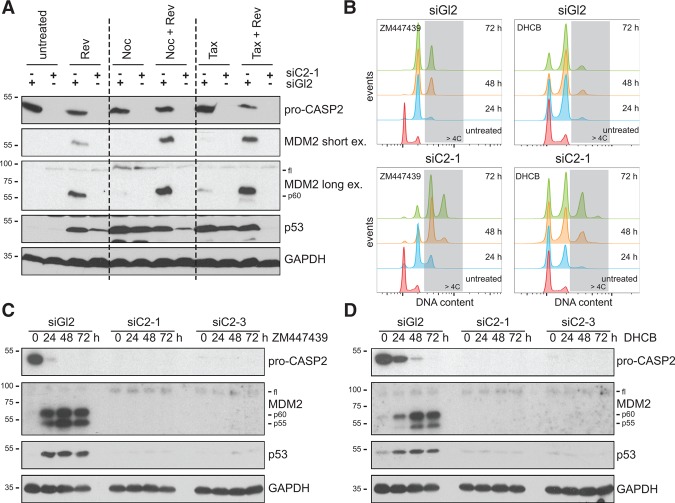
Caspase-2 constrains polyploidization after cytokinesis failure by MDM2 processing, leading to p53 stabilization*.* (*A*) A549 cells transfected with the indicated siRNAs targeting either luciferase (Gl2) or Caspase-2 (C2) were treated for 24 h with reversine (Rev), nocodazole (Noc), or taxol (Tax) alone or in combination and processed for immunoblotting. (*B*–*D*) Following transfection with siRNAs, cells were treated with the indicated cytokinesis inhibitors for different times and processed for DNA content analysis in a flow cytometer (*B*) or, in parallel, for immunoblotting (*C*,*D*).

As only a few bona-fide Caspase-2 substrates are known, we first validated the appearance of cleavage products of HDM2/MDM2, reported to be generated in response to DNA damage (Oliver et al. 2011), as a possible readout for Caspase-2 activity (Supplemental Fig. S1A,B). MDM2 cleavage products were generated in a strictly Caspase-2-dependent fashion and became detectable by immunoblotting when A549 lung adenocarcinoma cells underwent mitosis in the presence of the spindle assembly checkpoint (SAC) inhibitor reversine, targeting MPS1 kinase (Fig. 1A; Supplemental Fig. S1C,D; Santaguida et al. 2010). Caspase-2 activation was found to be further enhanced in the additional presence of microtubule targeting agents (MTAs) nocodazole or taxol. In stark contrast, MTAs alone, arresting cells in mitosis, failed to trigger significant Caspase-2 activity, arguing against a role as an effector in MTA-induced cell death or cell cycle arrest, as suggested by others (Ho et al. 2008).

**Figure 2.**
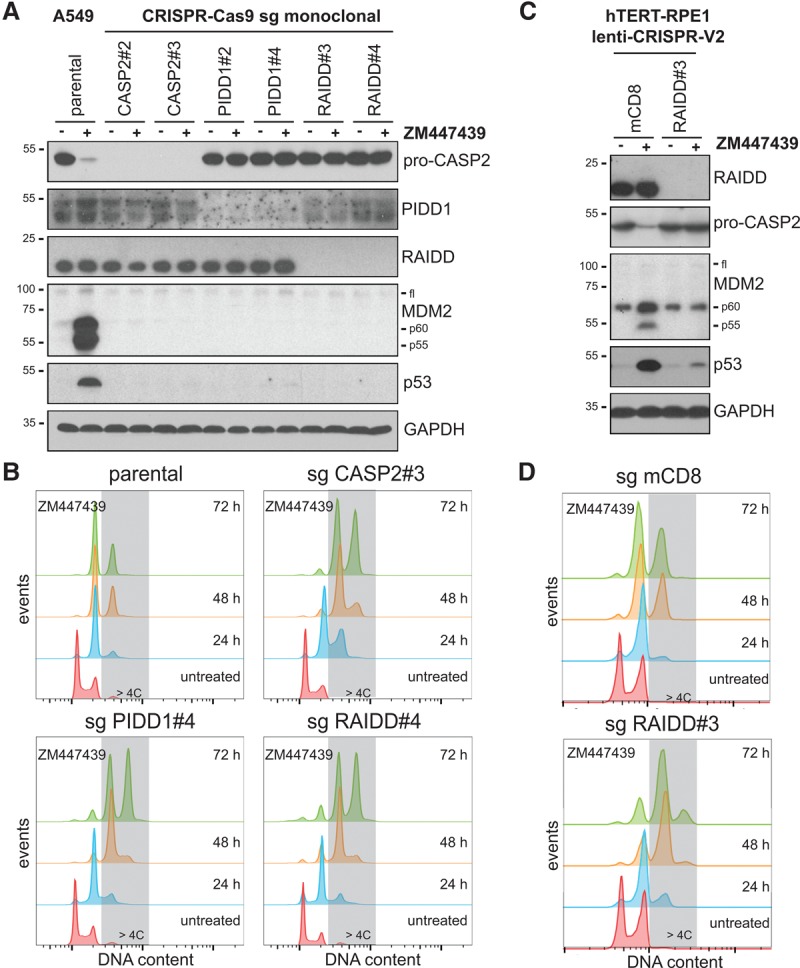
The PIDDosome is required to activate p53 after cytokinesis failure. A549 CASP2, PIDD1, or RAIDD knockout cells obtained using two different small guide RNAs (sgRNAs) were treated with ZM447439 for either 24 h and processed for immunoblotting (*A*) or up to 72 h and processed for DNA content analysis (*B*). hTERT-RPE1 cells were transduced with the indicated constructs targeting either mouse CD8 (mCD8) as a negative control or RAIDD and then treated with ZM447439 for either 24 h and processed for immunoblotting (*C*) or up to 72 h and processed for DNA content analysis (*D*).

While reversine blocks cytokinesis in only a fraction of A549 cells at the concentration used (Supplemental Fig. S1C,D), the penetrance of this phenotype is further enhanced by the addition of MTAs (Santaguida et al. 2010), suggesting that cytokinesis failure but not increased mitotic duration activates Caspase-2. Thus, we tested whether other means of inducing cytokinesis failure were equally potent in triggering Caspase-2 activation. Notably, perturbation of cytokinesis by Aurora B kinase inhibition (ZM447439), dihydro cytochalasin-B (DHCB) treatment, interfering with actin polymerization, or an siRNA targeting the RhoA-GEF ECT2 all triggered Caspase-2 activation, as monitored by the appearance of the MDM2 cleavage products (Fig. 1B–D; Supplemental Fig. S2). Cytokinesis failure also triggered p53 accumulation, p21 induction, and cell cycle arrest, all in the absence of notable cell death (Supplemental Fig. S2). Importantly, Caspase-2 activation was observed as early as 6 h following forced cytokinesis failure and resulted in cleavage of MDM2 at D367 (Supplemental Fig. S3), known to be sufficient for p53 stabilization (Oliver et al. 2011). Strikingly, depletion of Caspase-2 fully abrogated cleavage of MDM2, p53 accumulation, and cell cycle arrest of tetraploid cells (Fig. 1B–D). The same was observed when depleting PIDD1 or RAIDD by siRNA, although PIDD1 depletion had a milder impact (Supplemental Fig. S4A,B). However, CRISPR–Cas9-mediated deletion of Caspase-2, PIDD1, or RAIDD led to indistinguishable phenotypes (Fig. 2A,B), demonstrating that following cytokinesis failure, the PIDDosome acts as a functional unit to activate p53. Furthermore, we documented MDM2 cleavage in response to Aurora B kinase inhibition in various immortalized or transformed human cell lines, excluding a A549-specific phenomenon (Supplemental Fig. S4C). Finally, we confirmed RAIDD-dependent Caspase-2 activation also in telomerase immortalized noncancerous retinal pigmental epithelial cells (hTERT-RPE1) upon inhibition of Aurora B kinase, resulting in p53 induction to constrain polyploidization (Fig. 2C,D). Together, this demonstrates that our findings are not limited to human cancer cells.

### The PIDDosome is part of a genetically distinct p53 activation pathway engaged selectively after cytokinesis failure

**Figure 3.**
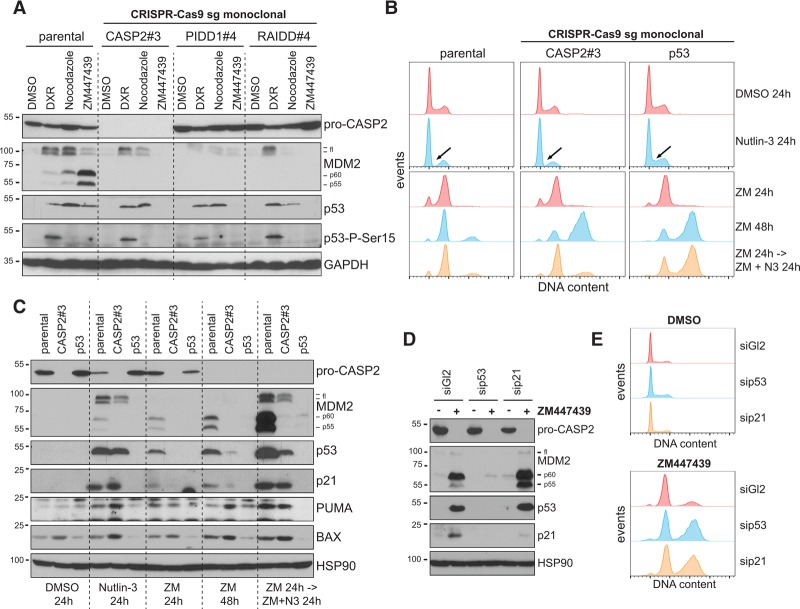
PIDDosome-activated p53 shares features with nongenotoxic activation via Nutlin-3 and functions via p21. (*A*) A549 knockout cells obtained using the indicated sgRNAs were treated with doxorubicin (DXR), nocodazole, or ZM447439 for 24 h and processed for immunoblotting. (*B*,*C*) A549 knockout cells obtained using the indicated sgRNAs were treated for the indicated times with Nutlin-3, ZM447439, or ZM447439 followed by the addition of Nutlin-3 and processed for DNA content analysis (*B*) or immunoblotting (*C*). (*D*,*E*) Following transfection with siRNAs, cells were treated with ZM447439 for 48 h and processed for immunoblotting (*D*) or DNA content analysis (*E*).

The contribution of the PIDDosome in stabilizing p53 appeared selective for cytokinesis failure, as its accumulation could still be observed in PIDDosome-deficient cells following DNA damage induced by doxorubicin or mitotic arrest triggered by nocodazole (Fig. 3A). Whereas DNA damage led to p53-Ser15 phosphorylation, extending the mitotic duration did not (Fig. 3A), consistent with the dispensability of the DDR kinases ATM, Chk1, and Chk2 in this pathway (Lambrus et al. 2016). Importantly, p53 stabilized by PIDDosome activation after cytokinesis failure also lacked phosphorylation on Ser15. Whereas 53BP1 and USP28 are required for p53 induction upon extended mitotic timing but dispensable for p53-mediated cell cycle arrest upon cytokinesis failure (Fong et al. 2016; Lambrus et al. 2016; Meitinger et al. 2016), we show that the PIDDosome is required for p53 stabilization upon cytokinesis failure but not upon prolonged mitotic timing or DNA damage. Of note, the latter two triggers do not rely on the generation of MDM2 cleavage fragments to activate p53 (Fig. 3A). Interestingly, transfection of an siRNA targeting the protein kinase LATS2, implicated in p53 activation upon cytokinesis failure, led to a measurable override of cell cycle arrest upon Aurora B kinase inhibition but without impinging on Caspase-2 activation or p53 stabilization (Supplemental Fig. S5), the latter contrasting published results (Ganem et al. 2014). Subcellular fractionation also revealed that the LATS2 substrate YAP maintained nuclear localization upon cytokinesis failure in A549 cells, suggesting that LATS2 kinase activity is not increased upon Aurora B kinase inhibition (Supplemental Fig. S5). Taken together, we identified a distinct and previously unnoticed mode of p53 activation with genetic requirements differing from the one of the DDR or the extension of mitotic timing.

### Caspase-2-deficient cells retain the ability to activate p53- and p21-mediated cell cycle arrest after failing cytokinesis

As PIDDosome-mediated p53 activation led to its accumulation devoid of Ser15 phosphorylation and therefore appeared similar to nongenotoxic p53 stabilization via pharmacological MDM2 inhibition (Thompson et al. 2004), we set out to test whether Caspase-2-deficient cells remain capable of activating p53 and arresting the cell cycle in response to Nutlin-3 upon cytokinesis failure. While p53 deficiency abrogated the responsiveness of cells to Nutlin-3, Caspase-2-deficient cells showed a clear loss of S-phase activity and cell cycle arrest, similar to that seen in parental cells (Fig. 3B). Furthermore, Caspase-2-deficient cells failing cytokinesis also responded to Nutlin-3 by showing a cell cycle arrest comparable with the one observed in parental cells, demonstrating that p53 function can be restored in those cells by using an MDM2 inhibitor.

In agreement with the finding that a fraction of p53 could be found in the nucleus upon PIDDosome-mediated stabilization (Supplemental Fig. S5), we observed transactivation of the cell cycle inhibitor p21 (Fig. 3C). Remarkably, knockdown of p21 by siRNA led to the same extent of excessive polyploidzation following cytokinesis failure that was observed upon p53 knockdown, showing that p21 is the most important mediator of cell cycle arrest upon failed cytokinesis (Fig. 3D,E). In contrast, only minor or no up-regulation of the proapoptotic p53 targets PUMA and BAX was observed upon either Nultin-3 treatment or cytokinesis failure. Surprisingly, when the same treatments were carried out in Caspase-2-deficient cells in which MDM2 cleavage was abrogated, an increased induction of PUMA and BAX was observed (Fig. 3C). This suggests that the presence of MDM2 cleavage fragments, particularly p60 that appeared to translocate into the nucleus (Supplemental Fig. S5) while retaining binding proficiency to p53 (Oliver et al. 2011), affects p53's selectivity for target genes.

### The PIDDosome limits polyploidization during liver organogenesis by controlling p53 activation

**Figure 4.**
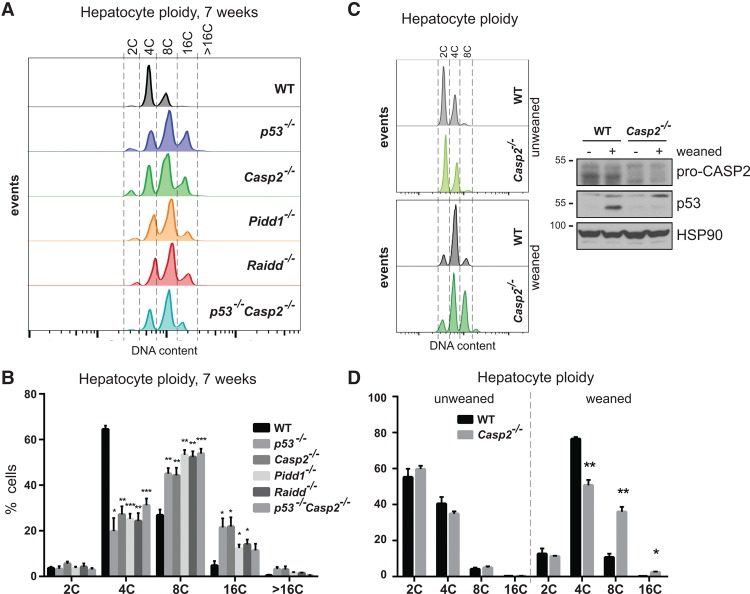
The PIDDosome constrains hepatocyte polyploidization in vivo via p53*.* (*A*) Hepatocytes were isolated by collagen perfusion of 7-wk-old mice of the indicated genotypes and subjected to DNA content analysis. (*B*) Quantification of the DNA content distribution for each genotype displayed in *A*. Bars represent mean percentages ± SEM. *n* = 3–5. (*C*) DNA content analysis and immunoblotting for hepatocytes obtained from animals of the indicated genotypes either unweaned (−) or weaned (+) at the age of 20 and 25 d, respectively. (*D*) Quantification of the DNA content distribution for each genotype displayed in *C*. *n* = 3. (*) *P* < 0.05; (**) *P* < 0.01; (***) *P* < 0.001 using unpaired Student's *t*-test.

To scrutinize these findings in vivo, we investigated the consequences of PIDDosome deficiency in a cell type known to undergo scheduled polyploidization due to blocked cytokinesis during organogenesis; namely, hepatocytes (Pandit et al. 2013). In young mice, hepatocytes proliferate as diploid cells (2C DNA content) until weaning, when the majority of mitoses culminates in incomplete cytokinesis, leading to tetraploidization (4C). Ploidy can further increase in a fraction of the cells with age, reaching 8C or even 16C DNA content in steady state. Hepatocyte ploidy can be enhanced even further during regeneration upon liver injury, and this ploidy increase as well as subsequently arising aneuploidy are thought to serve as a source of genetic variation, allowing swift adaptation to hepatotoxic stress (Pandit et al. 2013). Previous work has shown that p53 restrains this gradual increase in ploidy by reducing the proliferative potential of hepatocytes failing cytokinesis (Kurinna et al. 2013; Ganem et al. 2014). Accordingly, we observed significantly increased ploidy in hepatocytes isolated from *p53*^−/−^ animals. Strikingly, deficiency of each PIDDosome subunit mirrored the *p53*-null phenotype (Fig. 4A,B; Supplemental Fig. S6). While others have shown that LATS2 and Hippo pathway components are activated in polyploid hepatocytes, an event that can contribute to p53 stabilization (Ganem et al. 2014), we provide strong genetic evidence for the notion that p53 function in constraining liver polyploidization is fully neutralized upon elimination of Caspase-2 alone, as double deficiency in both p53 and Caspase-2 did not further increase hepatocyte ploidy (Fig. 4A,B). Additionally, Caspase-2 deficiency impinged significantly on ploidy distribution only after weaning (Fig. 4C,D), a time when the first wave of polyplodization is known to happen (Pandit et al. 2013). In full support of our model, p53 protein stabilization could be observed upon weaning in hepatocytes from wild-type but not Caspase-2-deficient mice (Fig. 4C). Taken together, our findings support the notion that the PIDDosome is a key activator of p53 following cytokinesis failure.

### Extra centrosomes are necessary and sufficient to activate the PIDDosome

**Figure 5.**
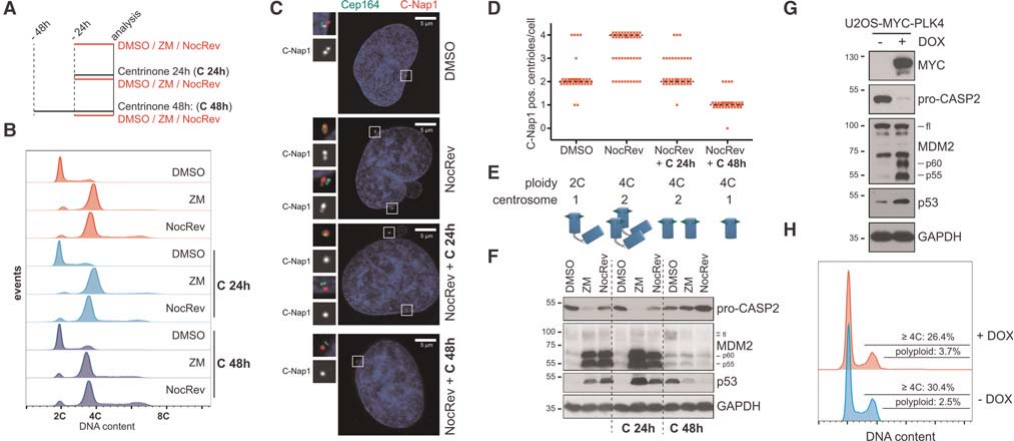
Extra centrosomes are necessary and sufficient for PIDDosome activation. (*A*) Scheme of the protocol used to generate polyploid cells with or without extra centrosomes. A549 cells were treated with either solvent control (DMSO), ZM447439 (ZM), or nocodazole plus reversine (NocRev) to promote cytokinesis failure in the absence or presence of 24 or 48 h of centrinone treatment. The combination of nocodazole plus reversine appeared best suited for faithful enumeration of centrioles by immunofluorescence (see below). Cells treated as in *A* were subjected to DNA content analysis (*B*), immunofluorescence (*C*,*D*), and immunoblotting with the indicated antibodies (*F*). (*D*) Scatter plot of cells stained as in *C* subjected to counting of C-Nap1-positive centrioles per cell. Fifty cells per condition were analyzed. Black dashed lines represent the median. (*E*) Summary of the features measured across the indicated treatments concerning ploidy and centrosomal content. The cartoon summarizes the typical centrosomal arrangement, with blue cylinders representing centrioles, ±green triangles representing the appendage structures decorating them, and markers (as Cep164) for mother centrioles. (*G*,*H*) U2OS-Trex-MYC-PLK4 cells were left untreated or treated with doxycycline (DOX) for 72 h and subjected to immunoblotting with the indicated antibodies (*G*) or DNA content analysis (*H*).

While this work identifies the PIDDosome as a regulator of cell cycle arrest following cytokinesis failure, it raises the question of which upstream signals can activate it in polyploid cells. Therefore, we aimed to uncouple increased ploidy from increased centrosome numbers, two main events occurring concomitantly with cytokinesis failure (Holland and Cleveland 2009). To this end, we used centrinone, a Polo-like-kinase 4 (PLK4) inhibitor, leading to centriole depletion by blocking their duplication during S phase (Wong et al. 2015). A 24-h cotreatment of A549 or U2OS cells with an agent triggering cytokinesis failure plus centrinone led to a measurable loss of centrioles without impinging on centrosome abundance (note that the two centrosomes per cell failing cytokinesis comprise either two centrioles each or only one centriole, depending on the presence or absence of centrinone, as opposed to one centrosome for each control cell) (Fig. 5A–E). This manipulation left the activation of the PIDDosome and therefore p53 unperturbed (Fig. 5F; Supplemental Fig. S7). In stark contrast, pretreatment of cells with centrinone for 24 h before addition of reagents inhibiting cytokinesis for an additional 24 h, leading to an effective centrosome reduction to one per tetraploid cell having failed cytokinesis, abrogated PIDDosome activation despite a notable increase in ploidy (Fig. 5A–F; Supplemental Fig. S7). Thus, doubling the centrosome number appeared most critical to activate the PIDDosome following cytokinesis failure. Conversely, the inducible overexpression of PLK4 in a U2OS derivative, which leads to centriole overduplication and therefore extra centrosomes (Kleylein-Sohn et al. 2007), triggered activation of the PIDDosome and p53 accumulation without concomitantly increasing cellular ploidy (Fig. 5G,H). Hence, we conclude that supernumerary centrosomes and not the increased ploidy trigger PIDDosome-dependent Caspase-2 activation.

To assess the effects of extra centrosomes on cell proliferation in the context of cytokinesis failure, a situation deemed more relevant for cancerogenesis, we monitored A549 cells forced to fail cytokinesis for up to 6 d. A sustained increase in DNA content could be observed until day 4, at which time the modal ploidy appeared to be 16C, but only a minor pool of cells could increase their ploidy further to 32C until day 6 (Supplemental Fig. S8). Remarkably, when cytokinesis failure was induced by either Aurora B inhibition or DHCB followed by release into fresh medium, up to 98% of Caspase-2-deficient cells underwent tripolar or tetrapolar anaphases, causing massive chromosomal segregation errors, as revealed by live-cell imaging (Supplemental Fig. S9). While other cell types appear more proficient in inducing clustering of extra centrosomes during mitosis and may display some degree of proliferation in the presence of polyploidy and extra centrosomes (Kwon et al. 2008; Holland et al. 2012), our finding documents that Caspase-2 deficiency per se is not sufficient to allow continued proliferation of polyploid cells carrying extra centrosomes.

### The PIDDosome responds to extra mature centrosomes

**Figure 6.**
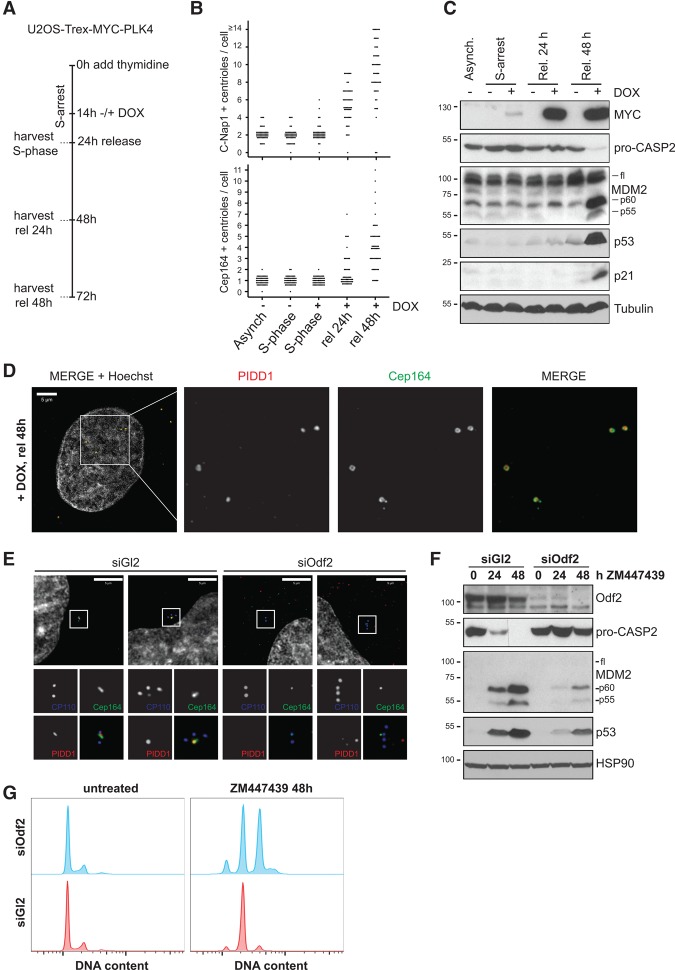
Extra mother centrioles activate the PIDDosome. (*A*) Scheme of the protocol used to time-resolve the appearance of extra centrioles at different maturation stages and PIDDosome activation. U2OS-Trex-MYC-PLK4 cells were arrested in S phase with thymidine. After 14 h, they were either left untreated or induced for an additional 10 h with doxycyline (Dox). Cells were either processed for immunofluorescence (Supplemental Fig. S10A) or immunoblot directly during S arrest or released for 24 and 48 h. (*B*) Scatter plot for the abundance of C-Nap1-positive and Cep164-positive centrioles assessed in 50 individual cells. (*C*) PIDDosome activation was followed by immunoblot analysis using the indicated antibodies. (*D*) Localization of PIDD1 at extra mother centrioles generated by PLK4 overexpression. (*E*–*G*) A549 cells transfected with the indicated siRNAs were subjected to immunofluorescence with the indicated antibodies (*E*) or treated with ZM447439 for the indicated times and subjected to either immunoblotting (*F*) or DNA content analysis (*G*).

Finally, we aimed to resolve the timing of PIDDosome activation in relation to the appearance of extra centrioles and hence centrosomes upon PLK4 overexpression. To this end, cells that underwent centriole overduplication during S-phase arrest were allowed to re-enter the cell cycle and were analyzed 24 or 48 h later (Fig. 6A). As expected, 24 h after resuming proliferation, the vast majority of the cells displayed excessive C-Nap1-positive centrioles, a marker for successful centriole disengagement and thus a marker for passage through mitosis (Wang et al. 2011). Forty-eight hours after the release, an increase in mother centrioles decorated by the distal appendage marker Cep164 (Graser et al. 2007), indicating full maturation of newly generated centrioles after two or more cell divisions, also became visible in most cells (Fig. 6B; Supplemental Fig. S10A). Strikingly, PIDDosome activity and p53 induction became apparent only 48 h after the release, demonstrating that PIDDosome activation correlates with the appearance of extra mother centrioles (Fig. 6C). Intriguingly, we noted that PIDD1 decorates mother centrioles in control cells (displaying maximally one PIDD1-positive centriole) (Supplemental Fig. S10B,C) and cells that had undergone centrosome overduplication, showing positivity for all excessive mother centrioles generated subsequent to PLK4 overexpression and repeated cell division (Fig. 6D).

**Figure 7.**
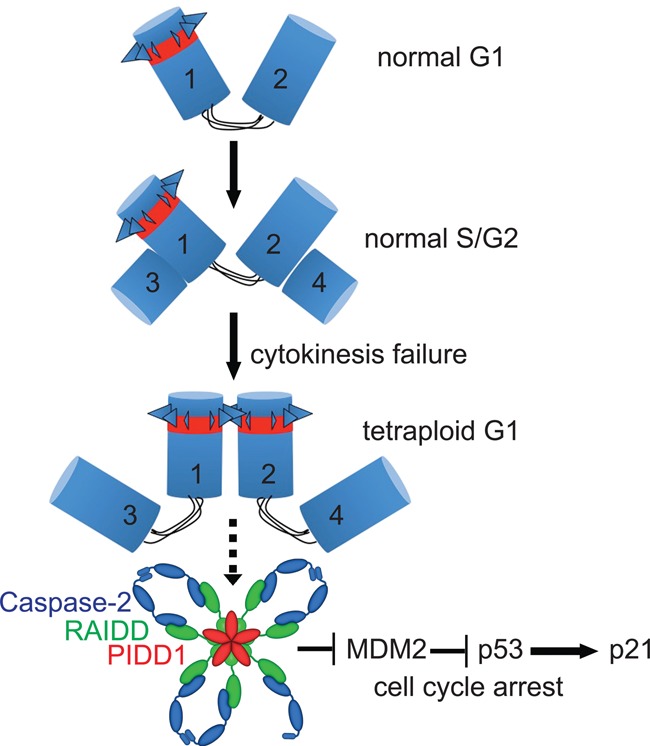
Proposed model of PIDDosome-mediated cell cycle arrest in response to supernumerary centrosomes. Cycling somatic cells carry one centrosome in G1, composed of one mother centriole (1)—decorated by appendages (triangles) and positive for PIDD1 (red)—and one daughter centriole (2). Both centrioles are connected by a flexible linker. During S phase, each centriole nucleates one procentriole (3 and 4). Upon mitotic traverse, the daughter centriole matures to mother and acquires appendages and PIDD1 positivity (2). In the absence of cytokinesis, the two mother centrioles (1 and 2) that would normally be inherited by the two daughter cells undergo clustering and promote PIDDosome assembly. This promotes Caspase-2 activation, which results in MDM2 cleavage at D367. The N-terminal fragment of MDM2 binds p53, leading to its stabilization and *p21* transcription-dependent cell cycle arrest.

To test whether the PIDDosome responds directly to the presence of extra mother centrioles or to secondary consequences of extra centrosomes, such as multipolar cell division, increased mitotic duration, or DNA damage, we depleted the centriolar protein Odf2/Cenexin, an established marker of mother centrioles, required for the formation of appendages and ciliogenesis. While appendages are the clearest distinguishing feature of mother centrioles over daughters, they are dispensable for faithful cell division (Ishikawa et al. 2005). Conceivably, Odf2 depletion not only impaired the localization of Cep164 to the centrosome, indicating a block in appendage formation, but also abrogated centrosomal localization of PIDD1 itself (Fig. 6E), while cell cycle distribution remained unperturbed (Fig. 6G). Consistent with a strict requirement for PIDD1 localizing to mature centrosomes for pathway initiation, Odf2 depletion by RNAi had a clear impact on the ability of cells to activate Caspase-2, cleave MDM2, up-regulate p53, and undergo cell cycle arrest upon cytokinesis failure (Fig. 6F,G). Hence, our data not only place the PIDDosome between supernumerary centrosomes and p53 but also suggest that PIDD1 is directly taking part in counting centrosomes, allowing cells to activate this pathway when they carry more than one mother centriole (Fig. 7).

## Discussion

Collectively, our work provides a possible explanation for the reported cellular and systemic defects caused by loss of Caspase-2, including increased aneuploidy and heightened tumor susceptibility upon oncogenic stress, metabolic dysfunction, or proteotoxic stress and premature aging. All of these features are well-known consequences of increased centrosomes, DNA ploidy, or aneuploidy (Davoli and de Lange 2011; Puccini et al. 2013; Donnelly et al. 2014). Furthermore, our data reveal a genuine physiological role of the PIDDosome in centrosome counting and uncover the missing link between supernumerary centrosomes and p53.

p53 appears the most relevant effector responding to mitotic defects, inducing cell cycle arrest or cell death following aberrant mitoses (Vitale et al. 2011). The triggers of mitotic defects can be very heterogeneous and include (1) DNA damage, occurring as either a consequence of sublethal caspase activation on extended mitosis (Orth et al. 2012) or a result of chromosome segregation defects (Janssen et al. 2011; Crasta et al. 2012); (2) the extension of the mitotic duration itself above a critical threshold (Uetake and Sluder 2010; Fong et al. 2016; Lambrus et al. 2016; Meitinger et al. 2016); (3) chromosome congression/segregation defects (Thompson and Compton 2010; Hinchcliffe et al. 2016); and (4) cytokinesis failure or centrosome amplification (Holland et al. 2012; Ganem et al. 2014). While the first three cues appear clearly distinct from each other, either requiring a definite set of genetic factors for p53 activation or associating with specific markers, it remained elusive whether the presence of extra centrosomes suffices to trigger p53 activation or whether this occurs as a consequence of the resulting CIN. Here, we demonstrated that the PIDDosome is activated primarily in response to cytokinesis failure and particularly by the underlying centrosome amplification, thereby describing yet another genetically distinct mode of p53 activation to secure genomic integrity.

While the appearance of MDM2 cleavage products by immunoblotting appeared the most reliable readout for Caspase-2 activation throughout our study, it also presents a main limitation: being poorly compatible with p53 deficiency. MDM2 basal expression requires p53, and it was therefore not possible to rely on MDM2 cleavage to assess whether Caspase-2 can become activated in p53-null cells or whether p53 is needed in a feed-forward loop fueling Caspase-2 activation by promoting PIDD1 transactivation, as suggested by others in the context of DNA damage (Oliver et al. 2011). Our observation of the disappearance of the Caspase-2 pro-form in the absence of signs of apoptotic effector caspase activity, indicative for activating autoprocessing (Baliga et al. 2004), supports the notion that Caspase-2 activation upon cytokinesis failure is p53-independent (Fig. 3C,D). This suggests, in turn, that basal levels of PIDD1 protein that can be detected also in p53-deficient cells (Cuenin et al. 2008) are sufficient to promote PIDDosome activation in response to cytokinesis failure. Subsequently, MDM2 cleavage products representing the N-terminal portion 1–367 of full-length MDM2 or of a shorter isoform of MDM2 devoid of the first 49 amino acids (Supplemental Fig. S3C; Oliver et al. 2011) accumulate and appear sufficient to stabilize p53 protein. While the fact that p53 bound to catalytically inactive MDM2 can transactivate *p21* is consistent with a previous study (Itahana et al. 2007), our data suggest that this may not apply globally to all p53 targets, as the proapoptotic genes BAX and PUMA appear to be transactivated to a much lesser extent in the presence of MDM2 cleavage fragments (Fig. 3C). Together, these observations assign the PIDDosome and Caspase-2 an unanticipated prosurvival function upstream of p53 rather than the often postulated cell death function downstream from it (Bock et al. 2012; Fava et al. 2012).

Of note, previous work had implicated the protein kinase LATS2 as an upstream regulator of p53 in response to mitotic defects and particularly following cytokinesis failure (Aylon et al. 2006; Ganem et al. 2014). While it seems premature to exclude that LATS2 can contribute to the activity of the Caspase-2–p53–p21 signaling axis (e.g., by direct binding to MDM2 or its cleavage products and affecting their localization or function) (Aylon et al. 2006), our data strongly suggest that PIDDosome-activated Caspase-2 is an essential and nonredundant upstream activator of p53 in response to acute cytokinesis failure in several cell lines and in developing hepatocytes in vivo.

It remains to be pointed out that cells failing cytokinesis or experiencing centrosome overduplication that may overcome the PIDDosome-dependent cell cycle arrest will experience, at later time points, secondary problems that will ultimately perturb their growth; e.g., due to increased CIN and/or aberrant subsequent mitosis (such as in Supplemental Fig. S9). We expect that these events will signal to p53 independently of the PIDDosome, thereby providing a possible explanation for why mouse mutants lacking the PIDDosome are largely normal and not overtly cancer-prone unless further challenged (Manzl et al. 2012). However, despite the PIDDosome function being more restricted than that displayed by p53, Caspase-2 deficiency was shown recently to be sufficient to predispose mice to chemically induced liver cancer (Shalini et al. 2016). Whether the observed Caspase-2 tumor suppressor function in this context requires the PIDDosome and how this relates to the increased hepatocyte ploidy described here need to be addressed experimentally.

While the fact that cell division failure in the presence of nocodazole still allows PIDDosome activation suggests that the pathway does not respond to varying levels of microtubule nucleation, we report the intriguing observation that PIDD1 localizes to mother centrioles throughout unperturbed interphase and decorates all extra mother centrioles when present (Fig. 6; Supplemental Fig. S10). Although this phenomenon per se does not provide functional insight, we present several lines of evidence that corroborate the notion that increasing the abundance of mother centrioles is the actual cue monitored by PIDD1. First, while normally cycling diploid cells invariably carry one mother centriole throughout interphase, cytokinesis failure, known to lead to the duplication of mother centrioles, triggers activation of the PIDDosome (Figs. 1, 2, 4). Second, when cytokinesis inhibitors are applied concomitantly to PLK4 inhibition, depletion of one centriole per centrosome does not affect pathway activation, but reducing the number of mother centrioles clearly does (Fig. 5A–F). Third, PLK4 overexpression leads to PIDDosome activation only at a time when excessive procentrioles have matured into mother centrioles (Fig. 6B,C). Thus, increasing either procentrioles or daughter centrioles does not suffice to activate the PIDDosome pathway, while increasing the number of mother centrioles clearly does (Fig. 6B,C). Finally, genetic inactivation of mother centriole identity by depletion of Odf2 impinges negatively on PIDD1 localization to the centrosome and simultaneously on the ability of the PIDDosome to become activated in response to cytokinesis failure.

Despite the fact that the available antibodies for RAIDD and Caspase-2 did not allow us to stain those proteins in immunofluorescence, we anticipate, based on the evidences discussed above, that the signal activating the PIDDosome after cytokinesis failure originates locally at the two mother centrioles (Fig. 7). While the responsiveness to mother centrioles provides an apparently convenient way to distinguish a G2 cell from a G1 cell following cytokinesis failure, thereby constraining PIDDosome activation to this latter circumstance, we presently do not know how PIDD1 can count mother centrioles or what cues then trigger PIDDosome assembly. As we observed that extra centrosomes cluster in interphase following cytokinesis failure (data not shown), it is tempting to speculate that PIDD1, immobilized on both centrosomes, is forced to undergo a conformational change, analogous to what is observed during apoptosome or inflammasome assembly, which allows it to serve as a scaffold for RAIDD, which then recruits Caspase-2, thereby promoting its autoprocessing and activation.

## Materials and methods

### Cell culture

Cell lines were cultured in DMEM (PAA Laboratories, E15-009; or Sigma-Aldrich, D5671) supplemented with 10% fetal bovine serum (FBS) (PAA Laboratories, A15-151), 1% L-glutamine (PAA Laboratories, M11-004), 100 U/mL penicillin, and 100 µg/mL streptomycin (PAA Laboratories, P11-010). Cells were incubated at 37°C with 5% CO_2_.

### Drug treatments and synchronization procedures

The following drugs were used alone or in combination: 0.5 µM reversine (Enzo Life Sciences, BML-SC104), 100 nM nocodazole (Sigma-Aldrich, M1404), 100 nM taxol (Sigma-Aldrich, T7191), 100 ng/mL doxorubicin (Sigma-Aldrich, D1515), 2 μM ZM447439 (Selleck Chemicals, S1103), 4 μM DHCB (Sigma-Aldrich, D1641), 125 nM centrinone (Wong et al. 2015), 2 mM thymidine (Sigma-Aldrich, T1895), 1 μg/mL doxocycline (Sigma-Aldrich, D9891), and 5 μM nutlin-3 (Sigma-Aldrich, N6287).

### Molecular cloning

Vectors for the generation of CRISPR–Cas9-mediated loss-of-function cell lines were made using the lentiCRISPR version 2 backbone (a gift from Feng Zhang; Addgene plasmid number 52961) (Sanjana et al. 2014). Oligonucleotides yielding small guide RNAs (sgRNAs) targeting the genes of interest were designed using CRISPR Design (http://crispr.mit.edu). The sequences are in the Supplemental Material. Cloning was performed according to the Feng Zhang protocol available at http://www.addgene.org. All plasmids were sequence-verified.

### Generation of cells lacking PIDDosome components

A549 cells were incubated with lentiviral supernatants generated using the plasmids described above and were selected for 3 d using 500 ng/mL puromycin (Sigma Aldrich, P9620). To obtain monoclonal lines, cells were seeded in 96-well plates to a density of 0.2 cells per well and incubated for 3 wk. Clones were further expanded and characterized for protein depletion by immunoblotting. To exclude clonal artifacts, at least two clones for each individual construct were analyzed for the ability to undergo polyploidization in response to ZM447439; one such clone was characterized further. hTERT-RPE1 cells were incubated with lentiviral supernatants as for A549 cells. Polyclonal (bulk) lines were used directly in the experiments.

### siRNA-mediated protein depletion

siRNA (40 nM) was premixed with 2 µL/mL Oligofectamine (Life Technologies, 12252-011) in Optimem (Life Technologies 31985-054) and incubated for 20 min at room temperature before transfection of the cells. Transfection took place 48 h before the beginning of drug treatment for various times (e.g., using ZM447439). Untreated/untransduced cells were harvested simultaneously with those treated/transduced for 24 h (i.e., after 72 h of total siRNA transfection).

### Cell lysis and immunoblotting

Cells were harvested by trypsinization. Samples were lysed in 50 mM Tris at pH 8.0, 150 mM NaCl, 0.5% NP-40, 50 mM NaF, 1 mM Na_3_VO_4_, 1 mM PMSF, one tablet protease inhibitors= (EDTA-free; Roche) per 10 mL, and 30 μg/mL DNase I, (Sigma Aldrich). Protein concentration was measured by Bradford analysis (Bio-Rad, 500-0006). Total protein (80–100 µg) was resolved by SDS-PAGE. Proteins were electroblotted on Amersham Hybond-ECL nitrocellulose membranes (GE Healthcare) using a wet transfer system (Bio-Rad).

### Statistical analysis

Statistical relevance (Fig. 3) was assessed using unpaired Student's *t*-test in Microsoft Excel. The histograms displayed were generated using Prism Graphpad software.

### Immunofluorescence microscopy

Cells were grown on glass coverslips (Hugo Sachs Elektronik, 64-0713) and washed once with PBS before fixation with absolute methanol for at least 10 min at −20°C. To maximize centriole visualization and allow visual counting, cells displayed in Figure 6, B and D, and Supplemental Figure S10A were treated for 1 h with 3.2 µM nocodazole before fixation. Samples were stored in methanol at −20°C or immediately stained. Stained cells were mounted in Mowiol, and images were acquired at room temperature on a SP5 confocal microscope (Leica) using a glycerol immersion objective (HCX plan apo 63×, NA 1.3); the recording software used was LASAF 2.7.3 (Leica). Images were deconvolved with Huygens Professional deconvolution and analysis software (Scientific Volume Imaging) and exported with Fiji to obtain maximum intensity projections of *z*-stacks that were further processed with Adobe Photoshop (adjusting contrast and brightness).

### DNA content analysis

Cell suspensions were washed with PBS and fixed with 70% ethanol in PBS for ≥10 min at −20°C. After fixation, cells were sedimented by centrifugation at 800*g* for 2 min, washed twice with PBS, and incubated with 40 µg/mL propidium iodide for 30 min at 37°C in the presence of RNase A. The stained cells were analyzed directly in a flow cytometer (LSR-Fortessa, BD Biosystems). Primary data were analyzed quantitatively, excluding doublets, using FlowJo (FlowJo, LLC).

### Primary hepatocyte isolation

Primary mouse hepatocytes were isolated by a two-step collagenase perfusion. Briefly, 7-wk-old mice were anesthetized (ketamine/xylasole intraperitoneally), the vena cava inferior was canulated and clamped below the heart, and the portal vein was incised. The liver was perfused with 70 mL of prewarmed (37°C) perfusion buffer (0.14 M NaCl, 6.7 mM KCl, 10 mM HEPES, 0.1 mM EGTA at pH 7.4) at 7 mL/min and 40 mL of collagenase buffer (66.7 mM NaCl, 6.7 mM KCl, 4.7 mM CaCl_2_*2H_2_O, 10 mM HEPES at pH 7.45) containing 25 µg/mL Liberase (Roche) at 2.5 mL/min at 37°C. The liver was gently disrupted in ice-cold L-15 medium (Leibowitz, Sigma), and the obtained cell suspension was passed through a 100-µm cell strainer. Viable hepatocytes were enriched by three subsequent low-speed centrifugations (30*g* for 3 min) in DMEM (Sigma) supplemented with 10% FCS and 2 mM L-glutamine. In order to determine the DNA content, 3 × 10^5^ hepatocytes were fixed in 70% ethanol. Cells were stained with 40 µg/mL propidium iodide in the presence of RNase A and analyzed on an LSR-Fortessa flow cytometer (BD Biosystems).

## Supplementary Material

Supplemental Material
