# Supplementary material for: The PIDDosome activates p53 in response to supernumerary centrosomes
Source: Genes Dev. 2017 Jan 1;31(1):34–45. doi: 10.1101/gad.289728.116 (PMC5287111; doi:10.1101/gad.289728.116)
Supplement: Supplemental Material [file supp_31_1_34__index.html]

Supplemental Material 

# The PIDDosome activates p53 in response to supernumerary centrosomes

## Supplemental Material

**Files in this Data Supplement:**

- Supplemental Material.pdf
