# Supplementary material for: The PIDDosome activates p53 in response to supernumerary centrosomes
Source: Genes Dev. 2017 Jan 1;31(1):34–45. doi: 10.1101/gad.289728.116 (PMC5287111; doi:10.1101/gad.289728.116)
Supplement: Supplemental Material [file supp_31.1.34_Supplemental_Material.pdf]

Supplementary figure legends:

Fig.S1 Mouse (m)Pidd1-CC overexpression triggers the appearance of MDM2 p55/p60 cleavage products in a RAIDD and Caspase-2-dependent manner. (A) A549 cells stably expressing a reverse transactivator (rtTA) were transfected with the indicated siRNAs for 48h, followed by transduction with mPIDD-CC WT or mPIDD-CC carrying the point mutation corresponding to human L828E, known to interfere with RAIDD binding (Park et al., 2007). 4h after the transduction, transgene expression (traceable in both cases by IRES-GFP) was induced for another 20h with Doxocycline. Cells were processed for immunoblotting using the indicated antibodies. * indicates signal generated by the GFP antibody that has been probed prior to RAIDD. (B) A549-rtTA cells were transfected with siRNAs targeting Luciferase (Gl2), Caspase-2 (C2-1), Caspase-9 (C9) or Caspase-8 (C8) for 48h, followed by transduction with mPIDD-CC and addition of Doxocycline, as described in (A). Cells were processed for immunoblotting with the indicated antibodies. (C-D) A549 cells treated with the indicated drug concentrations for 24h were processed for immunoblotting (C) or DNA content analysis (D), showing that Reversine, when used as in the manuscript at 500 nM, causes cytokinesis failure in a fraction of the cells and PIDDosome activation.

Fig.S2 Cytokinesis failure triggers Caspase-2 activation and p53 stabilization in the absence of cell death. A549 cells were treated with ZM447439 for different times and processed for immunoblotting (A), for DNA content analysis (B) or for DNA content analysis simultaneously to assessment of the proliferative behaviour using a Ki-67 staining and analysis in a flow cytometer (C). (D-E) Cells were transfected with siRNAs targeting targeting luciferase (Gl2) or ECT2 and processed for DNA content analysis in a flow cytometer (D) or for immunoblotting after various times (E). (F-G) Cells were treated with DHCB for different times and processed for DNA content analysis in a flow cytometer (F), or immunoblotting (G).

Fig.S3 Caspase-2 triggers cleavage of MDM2 at D367 at ≤ 6h following cytokinesis failure. (A) A549 cells were either left asynchronous (Asynch) or pre-synchronized in S-phase with thymidine, followed by release in the presence of Nocodazole for 12h. Mitotic cells were harvested by selective shake-off (Noco) and released in the presence of either solvent control (DMSO) or ZM447439 for various times. Samples were processed for immunoblotting with the indicated antibodies. (B) Cells were

either left asynchronous or synchronized by double thymidine arrest, followed by release in the presence of either solvent control (DMSO) or ZM447439 for various times. Samples were processed for immunoblotting with the indicated antibodies. Note that the majority of cells enter G1 approximately 12h after release according to parallel DNA content analysis (not shown). (C) Cells were left untreated or were transfected with MYC-tagged MDM2, either in its wild type form or carrying the indicated point mutations rendering them caspase-cleavage resistant. DMSO or ZM447439 treatment was performed 4h after the transfection for an additional 24h prior to sample processing for immunoblotting.

Fig.S4 siRNAs targeting the PIDDosome and PIDDosome activation in various cell lines. Following transfections with siRNAs, cells were left untreated or were treated with ZM447439 for 48h and processed for immunoblotting (A) or for DNA content analysis in a flow cytometer (B). (C) The indicated cell lines have been treated for 0, 24 and 48h with ZM447439 and processed for immunoblotting with the indicated antibodies.

Fig.S5. Assessment of Hippo and PIDDosome pathway interdependence. (A) A549 cells were either treated with solvent control (DMSO) or with ZM447439 for 24h and whole cell lysates (WCL) were harvested or fractionated in cytoplasmic (cyto) and nuclear components and subjected to immunoblotting. (B-C) Following transfections with siRNAs, A549 cells were left untreated or were treated with ZM447439 for 48h and processed for immunoblotting (B) or for DNA content analysis in a flow cytometer (C).

Fig.S6. Gating strategies for the analysis of hepatocyte ploidy. Example of gating strategy performed upstream of the hepatocyte data displayed in Fig. 4A-B. Cells were separated from debris first, followed by doublet-exclusion and quantitative assessment of ploidy distribution.

Fig.S7. PIDDosome activation requires the presence of extra centrosomes in U2OS cells. Cells treated as in (Fig. 5A) were subjected to DNA-content analysis (A) and in parallel to immunoblotting with the indicated antibodies (B).

Fig.S8 Long-term analysis of Caspase-2 deficient cells exposed to inhibition of cytokinesis. CASP2 knockout A549 cells obtained with the indicated sgRNA or parental A549 cells were treated with ZM447439 for the indicated times and processed for DNA content analysis in a flow cytometer (A) or for immunoblotting (B).

Fig.S9 Long-term live cell imaging analysis of Caspase-2 deficient cells exposed to inhibition of cytokinesis. (A) Movie stills from A549 parental cells or CASP2 knockout cells obtained with the indicated sgRNA, subjected to time-lapse video microscopy following exposure to solvent control (DMSO), ZM447439 and DHCB. Movies were acquired in the presence of siR-Hoechst to allow visualization of the chromatin. The cell shape was drawn before (first frame) and after (last frame) mitosis, based on phase contrast images (not shown). Time in hh:mm is indicated. Scale bar 10 μm. (B) Fate profiles of 50 cells treated as indicated followed from the first abortive mitosis (red) for 40h. (C) Movie stills from cells that have been treated for 24h with DHCB and released into fresh medium before imaging. While parental bi-nucleated cells did not traverse mitosis for the duration of imaging, CASP2 knockout displayed either bi-polar or tri/tetra-polar mitoses. (D) Quantification of the mitotic polarity distribution across to individual cells of the indicated genotypes exposed to the indicated treatments. (E) Box (interquartile range) and whisker (min to max) plots showing the elapsed time (min) between NEBD and anaphase for the 50 individual cells analysed in (D). (F) Example of cells exposed to DHCB for over 80h: cells were pre-treated with DHCB for 24h before imaging and video microscopy was performed in the continuous presence of DHCB. While parental bi-nucleated cells do not traverse mitosis for the duration of the movie, some CASP2 knockouts traverse two mitoses, showing that CASP2 deficient cells undergo up to three subsequent rounds of abortive mitosis in the 80h DHCB treatment window.

Fig.S10. Immunofluorescence staining, related to Figure 6. (A) Representative images of cells analyzed in the experiment shown in Figure 5A-C. (B) The anti-PIDD1 antibody used is specific in immunofluorescence: parental A549 and PIDD1 knockout derivatives obtained with the indicated CRISPR-Cas9 lentiviral constructs were stained in immunofluorescence with the indicated antibodies. Representative pictures are shown. (C) A549 and U2OS cells were stained in immunofluorescence with the indicated antibodies (2 centrioles for G1, 4 centrioles for S or G2 phases. Representative images are shown.

Supplementary methods

Cell Culture

MCF10A cells were cultured in DMEM/F12 (Invitrogen, 11330-032), supplemented with 5% horse serum (Invitrogen, 16050-122), 20 ng/ml EGF (Peprotech, AF-100-15), 0.5 mg/ml Hydrocortisone (Sigma Aldrich, H-0888), 100 ng/ml Cholera Toxin (Sigma Aldrich, C-8052), Insulin 10 µg/ml (Sigma Aldrich, I-1882), 100 U/ml penicillin and 100 µg/ml streptomycin (PAA laboratories, P11-010). All other cell lines were cultured in DMEM (PAA laboratories, E15-009 or Sigma-Aldrich, D5671) supplemented with 10% fetal bovine serum (FBS, PAA laboratories, A15-151), 1% L-glutamine (PAA laboratories, M11-004), 100 U/ml penicillin and 100 µg/ml streptomycin. Cells were incubated at 37 °C with 5% CO2. A549 (Haschka et al., 2015), U2OS (Sigl, Ploner, Shivalingaiah, Kofler, & Geley, 2014), U2OS-TRex-MYC-PLK4 (Kleylein-Sohn et al., 2007), hTERT RPE-1 (Sigl et al., 2014), Cal51 (Rashi-Elkeles et al., 2014) were previously described; MCF7 and MCF10A were a kind gift of Prof. H. Fiegl (Medical University of Innsbruck).

Cell Fractionation

Nuclear and cytoplasmic fractions were obtained according to (Suzuki, Bose, Leong-Quong, Fujita, & Riabowol, 2010). Purity of the fractions was then assessed using PARP1 and GAPDH immunoblotting, present exclusively in the nuclear and cytoplasmic fraction, respectively.

Synchronization Procedures

Synchronization in mitosis (Fig. S3A) was performed as follows: A549 cells were pre-synchronized by a single thymidine arrest (24h), followed by a release in fresh medium in the presence of Nocodazole for 12h. Mitotic cells were harvested by selective shake-off, washed twice in PBS and twice in medium and released in the presence of either DMSO or ZM447439. Synchronization at the G1-S boundary (Fig. S3B) was performed by 22 h arrest with thymidine, followed after 9 h by a second arrest of 17 h. For release cells were washed twice with PBS and fresh medium was added. Mitotic cells were harvested by selective shake off.

Cloning and site directed mutagenesis

The expression construct coding mPidd1-CC (Fig. S1) was generated using a mouse cDNA corresponding to *Pidd1*, isoform 1. The coding region from position 593-915 was amplified by PCR using high-fidelity Pfu-polymerase using the following oligonucleotides: GW-mPIDD-CC-fwd 5´ CAAAAAAGCAGGCTCCatgTCCTGGTACTGGCTCTGGTATACC, and GW-mPIDD-CC-rev 5´ CAAGAAAGCTGGGTctaGGCCTGTGCAGACTCTGG. The PCR product was cloned into pDONR207 (Invitrogen) using BP clonase reaction mix (Thermo Fisher Scientific, Vienna) and then recombined into the lentiviral vector pHR-tet-CMV-Dest-IRES-GFP using the GATEWAY™ technology (Invitrogen). The variant carrying the point mutation referred as L828E (corresponding to mouse L833E) was obtained by site directed Quickchange™ mutagenesis (Agilent), using the following oligonucleotides: mPIDD-L828E-fwd 5 AATTCAGGGATGACgagGATGGCCAGGTCCGAC, mPIDD-L828E-rev 5´ GTCGGACCTGGCCATCctcGTCATCCCTGAATTC. Oligonucleotides yielding sgRNAs were sgCASP2#2 fwd: 5´caccgAGGACTCACACACCGGAAAA, rev: 5´ aaacTTTTCCGGTGTGTGAGTCCTc; sgCASP2#3 fwd: 5´ caccgTGGTGAGCAACATATCCTCC, rev: 5´aaacGGAGGATATGTTGCTCACCAc; sgPIDD1#2 fwd: 5´caccgGCCGATAGCGGATGGTGATG, rev: 5´aaacCATCACCATCCGCTATCGGCc; sgPIDD1#4 fwd: 5´caccgGGCCCGGCGCTGCCGTGAAG, rev: 5´aaacCTTCACGGCAGCGCCGGGCCc; sgRAIDD#3 fwd: 5´caccgCGCTCACTTCGCCTGGAGCT, rev: 5´aaacAGCTCCAGGCGAAGTGAGCGc; sgRAIDD#4 fwd: 5´caccgCCAGCTCCAGGCGAAGTGAG, rev: 5´aaacCTCACTTCGCCTGGAGCTGGc; mCD8 fwd: caccgGCTGGGTGAGTCGATTATCC, rev 5' aaacGGATAATCGACTCACCCAGCc; sgp53 fwd: 5' caccgTCCATTGCTTGGGACGGCAA, rev: aaacTTGCCGTCCCAAGCAATGGAc.

Plasmid transfection

For plasmid transfections (Fig. S3C) 2.5 µg of plasmid DNA were transfected into A549 cells (500.000 per 6 cm plate) using Metafectene (Biontex) according to the manifacturer´s protocol. The following plasmids were employed: pCMV-MYC3-HDM2, a gift of Yue Xiong, Addgene plasmid 20935 (Zhang et al., 2003), pCMV-

MYC3-MDM2 D367E (Addgene 52057) and pCMV-MYC3-MDM2 D367A (Addgene 52058) were a gift of T. Jacks and T. Oliver (Oliver et al., 2011).

siRNA sequences

siRNA duplexes (purchased from Microsynth AG) were siC2-1 GCC CAA GCC UAC AGA ACA AdTdT, siC2-3 ACA GCU GUU GUU GAG CGA AdTdT, siC8 AAG AGU CUG UGC CCA AAU CAA dTdT, siC9 CCA GGC AGC UGA UCA UAG AdTdT, siECT2 GCA CUC ACC UUG UAG UUG AdTdT, siRAIDD GGG UUU CCA CUA GAC AUU AdTdT, siPIDD1-1 CAG ACU GUU CCU GAC CUC AGA dTdT, siPIDD1-2 CUG CUU UGU CUU CUA CUC GCA dTdT, siPIDD1-3 AGA CCU ACC UGG AGG AAG AdTdT, sip21 ACA AAG UCG AAG UUC CAU CdTdT, sip53 AGU AGA UUA CCA CUG GAG UCdTdT, siLATS2 GCA CGC AUU UUA CGA AUU CdTdT, siODF2 GAG GUC AAG AUG CAA AAA GGU dTdT.

Antibodies

The following antibodies were used in immunoblotting experiments: rabbit anti GAPDH (14C10 clone, Cell Signaling, 2118, 1:5000), rat anti CASP2 (clone 10C6, 1 µg/ml), rabbit anti p53 (used throughout the manuscript, FL-393, Santa Cruz Biotechnology, sc 6243, 1:200), mouse anti p53 (used in hepatocytes, 1C12, Cell Signaling, 2524, 1:500), mouse anti phosho-p53 Ser15 (clone 16G8, Cell Signaling, 9286, 1:500), mouse anti HSP90 (clone F8, Santa Cruz Biotechnology, sc-13119, 1:1000) mouse anti MDM2 (IF2, Thermo Fisher Scientific, MA1-113, 1:500), mouse anti CHK1 (clone 2G1D5, Cell Signaling, 2360, 1:1000), rabbit anti phospho-CHK1 Ser345 (Clone 133D3, Cell Signaling, 2348, 1:500), rabbit anti phospho-cdc2 Tyr15 (Cell Signaling, 9111, 1:500), mouse anti p21 (BD, 554262, 1:500), mouse anti MYC (9E10, 1 µg/ml), mouse anti Tubulin (Sigma-Aldrich, T6199, 1:5000), rabbit anti GFP (rabbit SG4.1, 1:1000), mouse anti RAIDD (clone 4B12, LSBio, LS-C179788, 1:1000), mouse anti CASP9 (Cell Signaling, 9508, 1:1000), rabbit anti CASP8 (R&D Systems, AF1650, 1:400), rabbit anti PARP1 (Cell Signaling, #9542, 1:1000), mouse anti CDC27 (clone 35/CDC27, 1:300, BD 610455), mouse anti PIDD1 (ENZO, ALX-804-837-C100, 1:500), rabbit ODF2 (Sigma, HPA001874, 1:1000), rabbit anti YAP antibody (Cell Signaling, 4912, 1:1000), rabbit anti BAX antibody (Cell Signaling, 2772, 1:1000), rabbit anti PUMA antibody (Cell Signaling, 4976, 1:1000), goat anti rabbit Ig/HRP (Dako, P0448, 25 ng/ml), rabbit anti mouse Ig/HRP (Dako, P0161,

0.13 µg/ml), rabbit anti rat IgG heavy chain/HRP (Jackson ImmunoResearch, discontinued, 1:10000). In immunofluorescence, the following antibodies were used: rabbit Cep164 (Graser et al., 2007) (1:500) rabbit CP110 (Schmidt et al., 2009) (1:500), mouse anti C-Nap1 (this study, see below, hybridoma supernatant 1:5), mouse PIDD1 (ENZO, ALX-804-837-C100, 1:500). Cep164 and CP110 antibodies were directly coupled to Alexa Fluor 488 and 555, respectively (Invitrogen), while anti PIDD1 and C-Nap1 antibodies were detected by goat anti-mouse antibodies coupled to either Alexa Fluor 488 (Fig. S7C), Alexa Fluor 568 (Fig. 4C and 5D) or Alexa Fluor 647 (Fig. 5E and S7A-B), all used 1:750 (Invitrogen). The DNA was stained with 1 µg/ml Hoechst 33342.

Generation of the C-Nap1 monoclonal antibody

Hybridoma cell lines producing monoclonal antibodies against C-Nap1 were generated following standard procedures. A recombinant fragment of human C-Nap1 (coding for amino acids 1988-2442) was expressed in E.coli, purified and injected into mice. The monoclonal antibody produced by the hybridoma cell line 150-230-1 is of the IgG1 subclass.

Mouse strains

Generation and genotyping of *p53*$^{-/-}$ (Lowe, Schmitt, Smith, Osborne, & Jacks, 1993)*, Casp2*$^{-/-}$(O'Reilly et al., 2002) *, Pidd1*$^{-/-}$ (Manzl et al., 2009) *and Raidd*$^{-/-}$ (Berube et al., 2005) mice have been described. All mice were maintained on a C57BL6 genetic background and used at the indicated age. Animal experiments were performed in line with Austrian legislation (BMWFW-66.011/0108-WF/V3b/2015).

Primary Hepatocyte Isolation

The two-step collagenase perfusion described in the main text was adapted from (Grompe, Jones, Loulseged, & Caskey, 1992; Seglen, 1976; Theurl et al., 2011).

Time-lapse video microscopy

Cells were seeded 30 h before imaging into Ibidi µ-Slide 8 Well coverslip (Cat. No. 80826) at a density of 20.000 cells per well. 4 h later cells were either left untreated

or treated with cytokinesis inhibitors. 2 h before imaging cells were washed with PBS twice and with Leibovitz 15 (LifeTechnologies, 21083-027) medium supplemented with 10 % FBS, 100 u/ml penicillin, 100 µg/ml streptomycin two more times. Cells were incubated in the same medium -/+ cytokinesis inhibitors in the presence of 2 µM SiR-Hoechst at 37°C (Lukinavičius et al., 2015). Imaging at multiple positions was performed every 5 min on a Leica DMI8 AF6000LX system equipped with Adaptive Focus Control, an HC PL FLUOTAR L 20x/0.40 dry objective, Hamamatsu Flash4.0 camera and a Lumencor Spectra x light engine. Lif files were processed with Fiji and the contrast of movie stills was adjusted in Adobe Photoshop CS6.

Supplementary References

Berube, C., Boucher, L. M., Ma, W., Wakeham, A., Salmena, L., Hakem, R., et al. (2005). Apoptosis caused by p53-induced protein with death domain (PIDD) depends on the death adapter protein RAIDD. *Proc Natl Acad Sci U S A*, *102*(40), 14314–14320. http://doi.org/10.1073/pnas.0506475102

Graser, S., Stierhof, Y. D., Lavoie, S. B., Gassner, O. S., Lamla, S., Le Clech, M., & Nigg, E. A. (2007). Cep164, a novel centriole appendage protein required for primary cilium formation. *J Cell Biol*, *179*(2), 321–330. http://doi.org/10.1083/jcb.200707181

Grompe, M., Jones, S. N., Loulseged, H., & Caskey, C. T. (1992). Retroviral-mediated gene transfer of human ornithine transcarbamylase into primary hepatocytes of spf and spf-ash mice. *Hum Gene Ther*, *3*(1), 35–44. http://doi.org/10.1089/hum.1992.3.1-35

Haschka, M. D., Soratroi, C., Kirschnek, S., Hacker, G., Hilbe, R., Geley, S., et al. (2015). The NOXA-MCL1-BIM axis defines lifespan on extended mitotic arrest. *Nat Commun*, *6*, 6891. http://doi.org/10.1038/ncomms7891

Kleylein-Sohn, J., Westendorf, J., Le Clech, M., Habedanck, R., Stierhof, Y. D., & Nigg, E. A. (2007). Plk4-induced centriole biogenesis in human cells. *Dev Cell*, *13*(2), 190–202. http://doi.org/10.1016/j.devcel.2007.07.002

Lowe, S. W., Schmitt, E. M., Smith, S. W., Osborne, B. A., & Jacks, T. (1993). p53 is required for radiation-induced apoptosis in mouse thymocytes. *Nature*, *362*(6423), 847–849. http://doi.org/10.1038/362847a0

Lukinavičius, G., Blaukopf, C., Pershagen, E., Schena, A., Reymond, L., Derivery, E., et al. (2015). SiR-Hoechst is a far-red DNA stain for live-cell nanoscopy. *Nat Commun*, *6*, 8497. http://doi.org/10.1038/ncomms9497

Manzl, C., Krumschnabel, G., Bock, F., Sohm, B., Labi, V., Baumgartner, F., et al. (2009). Caspase-2 activation in the absence of PIDDosome formation. *J Cell Biol*, *185*(2), 291–303. http://doi.org/10.1083/jcb.200811105

O'Reilly, L. A., Ekert, P., Harvey, N., Marsden, V., Cullen, L., Vaux, D. L., et al. (2002). Caspase-2 is not required for thymocyte or neuronal apoptosis even though cleavage of caspase-2 is dependent on both Apaf-1 and caspase-9. *Cell Death Differ*, *9*(8), 832–841. http://doi.org/10.1038/sj.cdd.4401033

Oliver, T. G., Meylan, E., Chang, G. P., Xue, W., Burke, J. R., Humpton, T. J., et al. (2011). Caspase-2-mediated cleavage of Mdm2 creates a p53-induced positive feedback loop. *Mol Cell*, *43*(1), 57–71. http://doi.org/10.1016/j.molcel.2011.06.012

Park, H. H., Logette, E., Raunser, S., Cuenin, S., Walz, T., Tschopp, J., & Wu, H. (2007). Death domain assembly mechanism revealed by crystal structure of the oligomeric PIDDosome core complex. *Cell*, *128*(3), 533–546. http://doi.org/10.1016/j.cell.2007.01.019

Rashi-Elkeles, S., Warnatz, H. J., Elkon, R., Kupershtein, A., Chobod, Y., Paz, A., et al. (2014). Parallel profiling of the transcriptome, cistrome, and epigenome in the cellular response to ionizing radiation. *Sci Signal*, *7*(325), rs3. http://doi.org/10.1126/scisignal.2005032

Schmidt, T. I., Kleylein-Sohn, J., Westendorf, J., Le Clech, M., Lavoie, S. B., Stierhof, Y. D., & Nigg, E. A. (2009). Control of centriole length by CPAP and CP110. *Curr Biol*, *19*(12), 1005–1011. http://doi.org/10.1016/j.cub.2009.05.016

Seglen, P. O. (1976). Preparation of isolated rat liver cells. *Methods Cell Biol*, *13*, 29–83.

Sigl, R., Ploner, C., Shivalingaiah, G., Kofler, R., & Geley, S. (2014). Development of

a multipurpose GATEWAY-based lentiviral tetracycline-regulated conditional RNAi system (GLTR). *PLoS One*, *9*(5), e97764. http://doi.org/10.1371/journal.pone.0097764

Suzuki, K., Bose, P., Leong-Quong, R. Y., Fujita, D. J., & Riabowol, K. (2010). REAP: A two minute cell fractionation method. *BMC Research Notes*, *3*(1), 294. http://doi.org/10.1186/1756-0500-3-294

Theurl, I., Schroll, A., Sonnweber, T., Nairz, M., Theurl, M., Willenbacher, W., et al. (2011). Pharmacologic inhibition of hepcidin expression reverses anemia of chronic inflammation in rats. *Blood*, *118*(18), 4977–4984. http://doi.org/10.1182/blood-2011-03-345066

Zhang, Y., Wolf, G. W., Bhat, K., Jin, A., Allio, T., Burkhart, W. A., & Xiong, Y. (2003). Ribosomal protein L11 negatively regulates oncoprotein MDM2 and mediates a p53-dependent ribosomal-stress checkpoint pathway. *Mol Cell Biol*, *23*(23), 8902–8912.

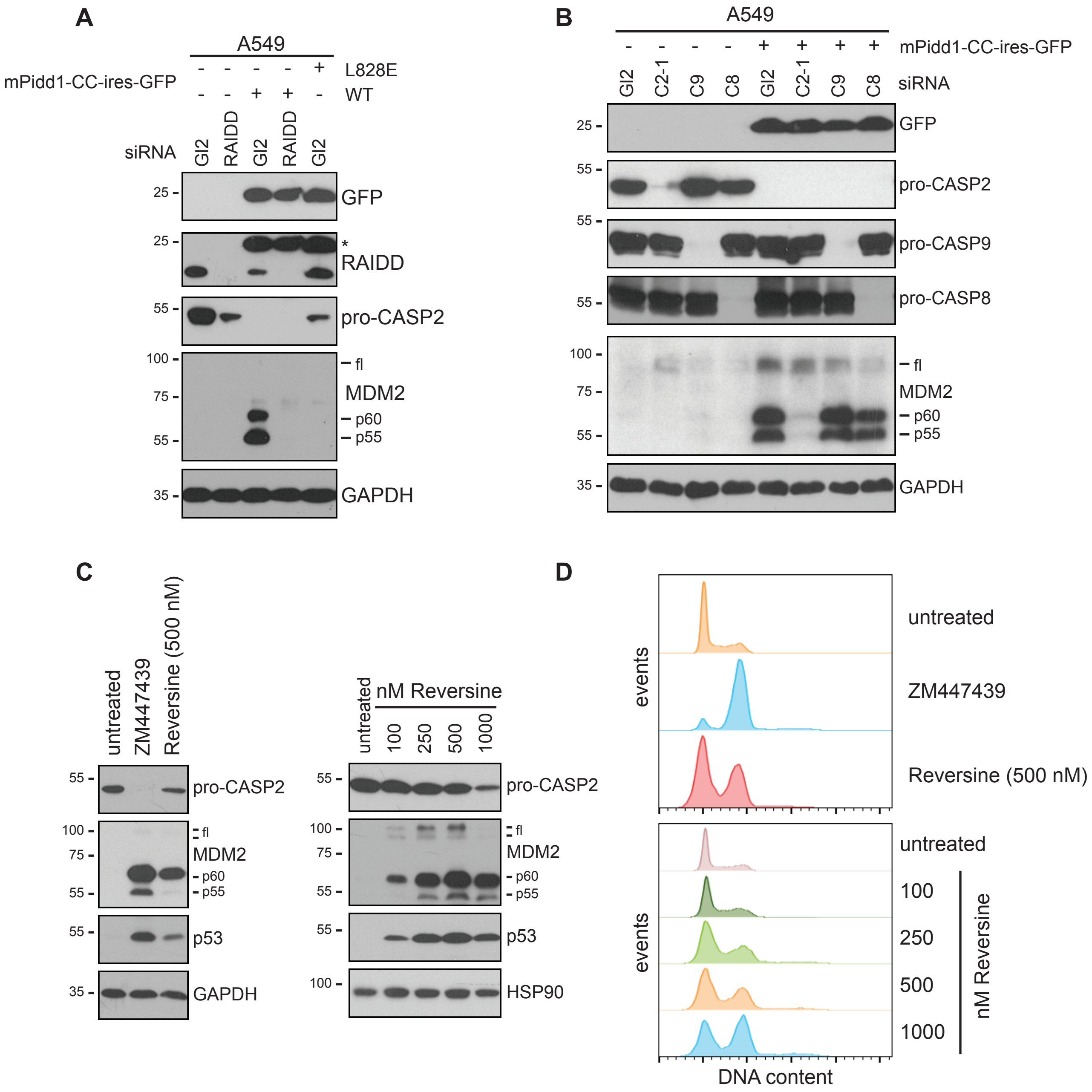
A
A549
mPidd1-CC-ires-GFP
L828E
WT
siRNA
Gl2
RAIDD
GFP
RAIDD
pro-CASP2
MDM2
fl
p60
p55
GAPDH
B
A549
mPidd1-CC-ires-GFP
siRNA
Gl2
C2-1
C9
C8
GFP
pro-CASP2
pro-CASP9
pro-CASP8
MDM2
GAPDH
C
untreated
ZM447439
Reversine (500 nM)
nM Reversine
100
250
500
1000
pro-CASP2
MDM2
p53
GAPDH
HSP90
D
events
untreated
ZM447439
Reversine (500 nM)
nM Reversine
DNA content

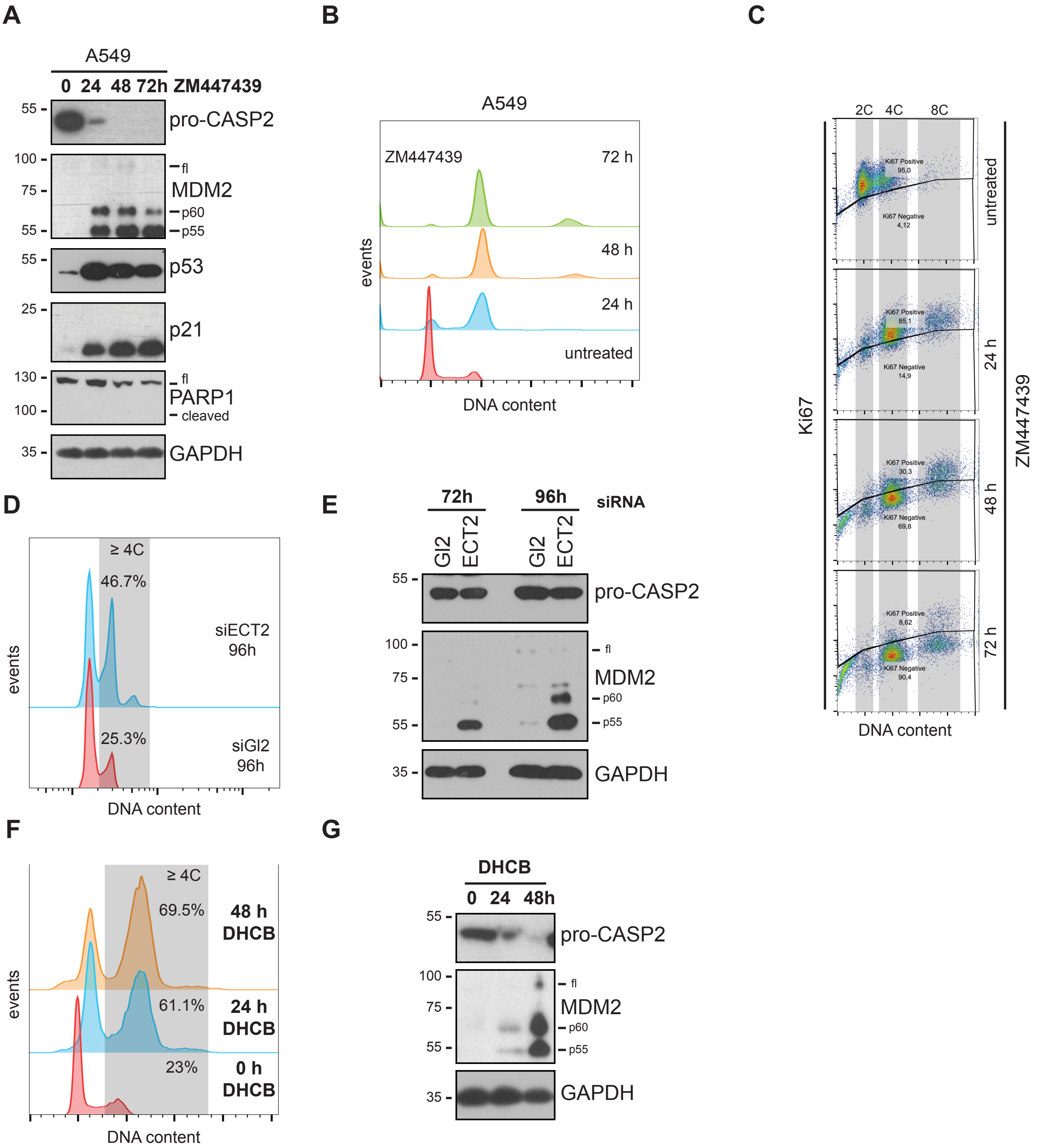
A
A549
0 24 48 72h ZM447439
55
pro-CASP2
100
75
55
fl
MDM2
p60
p55
55
p53
25
p21
130
100
fl
PARP1
cleaved
35
GAPDH
B
A549
ZM447439
72 h
48 h
24 h
untreated
events
DNA content
C
2C 4C 8C
Ki67 Positive 95,0
Ki67 Negative 4,12
Ki67 Positive 85,1
Ki67 Negative 14,9
Ki67 Positive 30,3
Ki67 Negative 69,8
Ki67 Positive 8,62
Ki67 Negative 90,4
untreated
24 h
48 h
72 h
ZM447439
Ki67
DNA content
D
≥ 4C
46.7%
siECT2 96h
25.3%
siGl2 96h
events
DNA content
E
72h 96h siRNA
Gl2 ECT2 Gl2 ECT2
55
pro-CASP2
100
75
55
fl
MDM2
p60
p55
35
GAPDH
F
≥ 4C
69.5%
48 h DHCB
61.1%
24 h DHCB
23%
0 h DHCB
events
DNA content
G
DHCB
0 24 48h
55
pro-CASP2
100
75
55
fl
MDM2
p60
p55
35
GAPDH

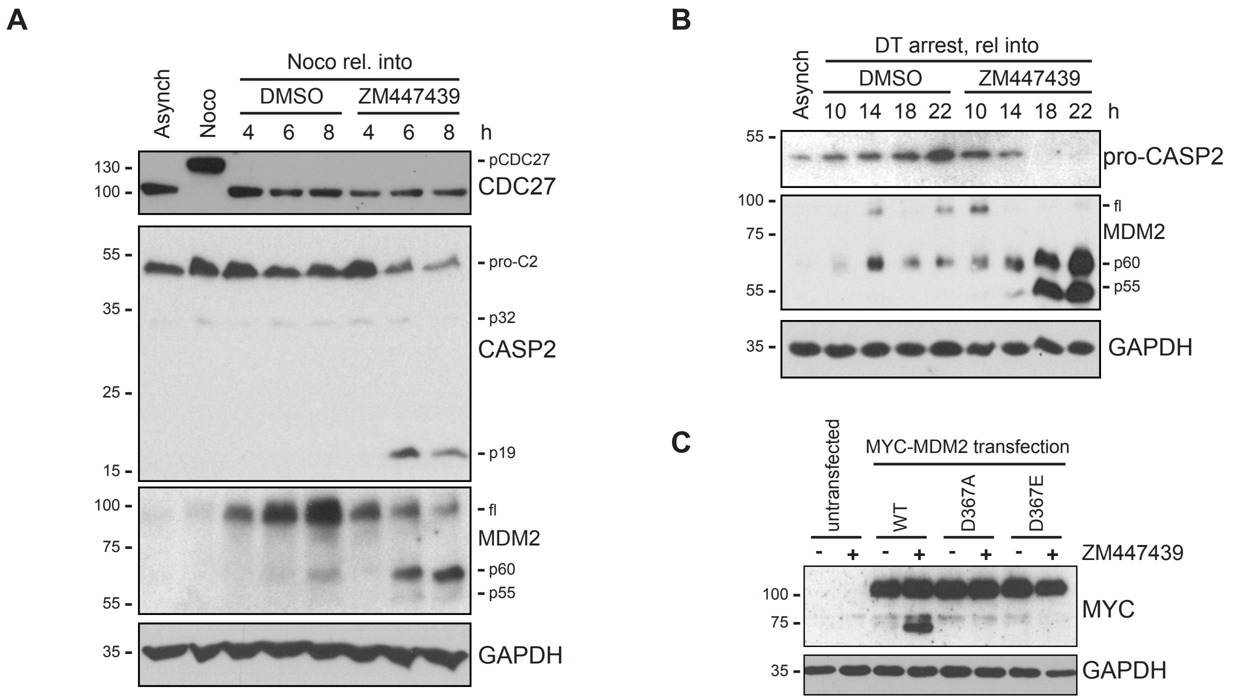
A
Noco rel. into
DMSO
ZM447439
Asynch
Noco
4
6
8
4
6
8
h
130
100
pCDC27
CDC27
55
35
25
15
pro-C2
p32
CASP2
p19
100
75
55
fl
MDM2
p60
p55
35
GAPDH
B
DT arrest, rel into
DMSO
ZM447439
Asynch
10
14
18
22
10
14
18
22
h
pro-CASP2
fl
MDM2
p60
p55
GAPDH
C
MYC-MDM2 transfection
untransfected
WT
D367A
D367E
ZM447439
MYC
GAPDH

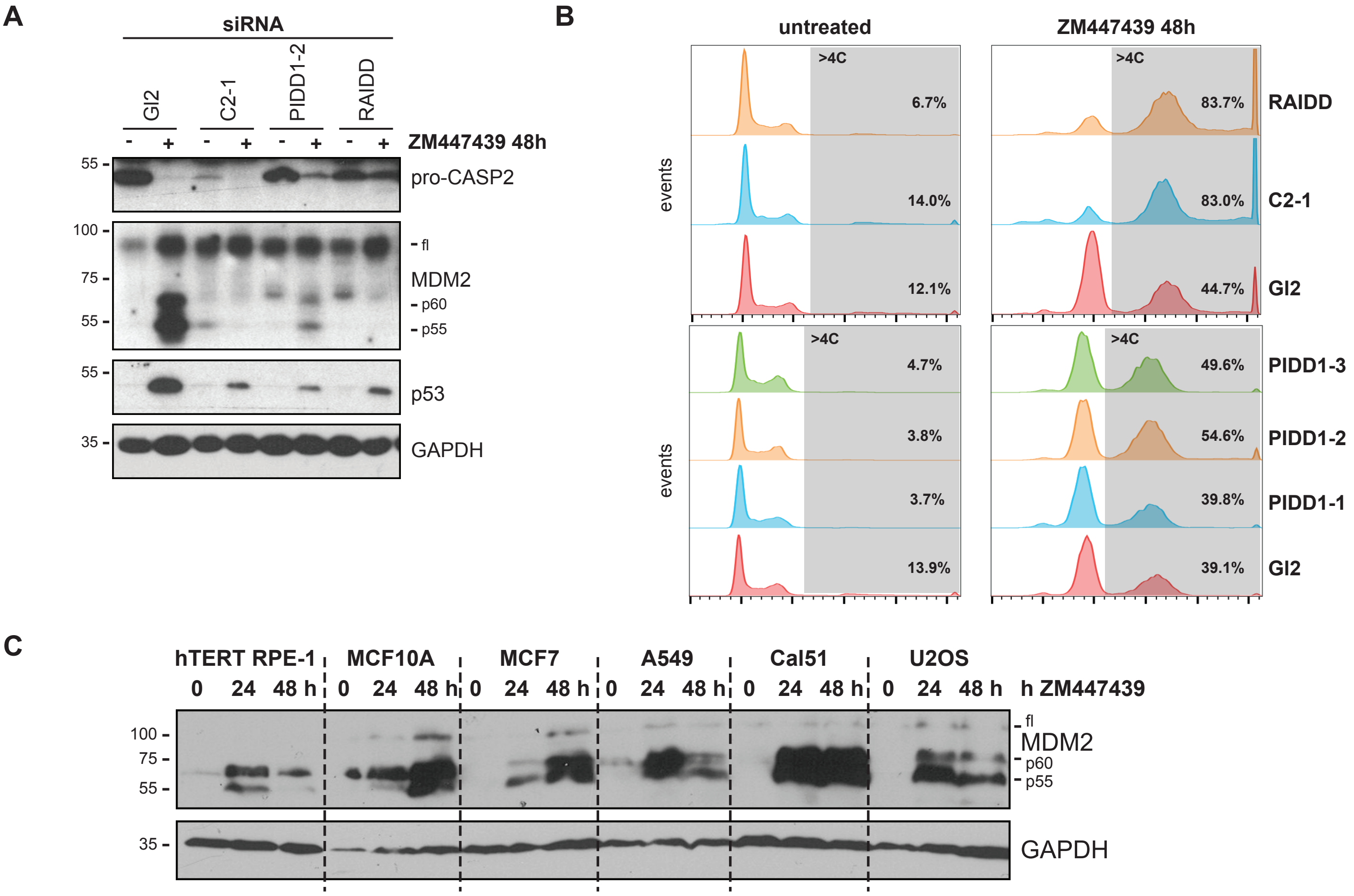
A
siRNA
Gl2
C2-1
PIDD1-2
RAIDD
ZM447439 48h
55
pro-CASP2
100
75
55
fl
MDM2
p60
p55
55
p53
35
GAPDH
B
untreated
ZM447439 48h
events
>4C
6.7%
83.7%
RAIDD
14.0%
83.0%
C2-1
12.1%
44.7%
Gl2
4.7%
49.6%
PIDD1-3
3.8%
54.6%
PIDD1-2
3.7%
39.8%
PIDD1-1
13.9%
39.1%
Gl2
C
hTERT RPE-1
MCF10A
MCF7
A549
Cal51
U2OS
0 24 48 h
h ZM447439
100
75
55
fl
MDM2
p60
p55
35
GAPDH

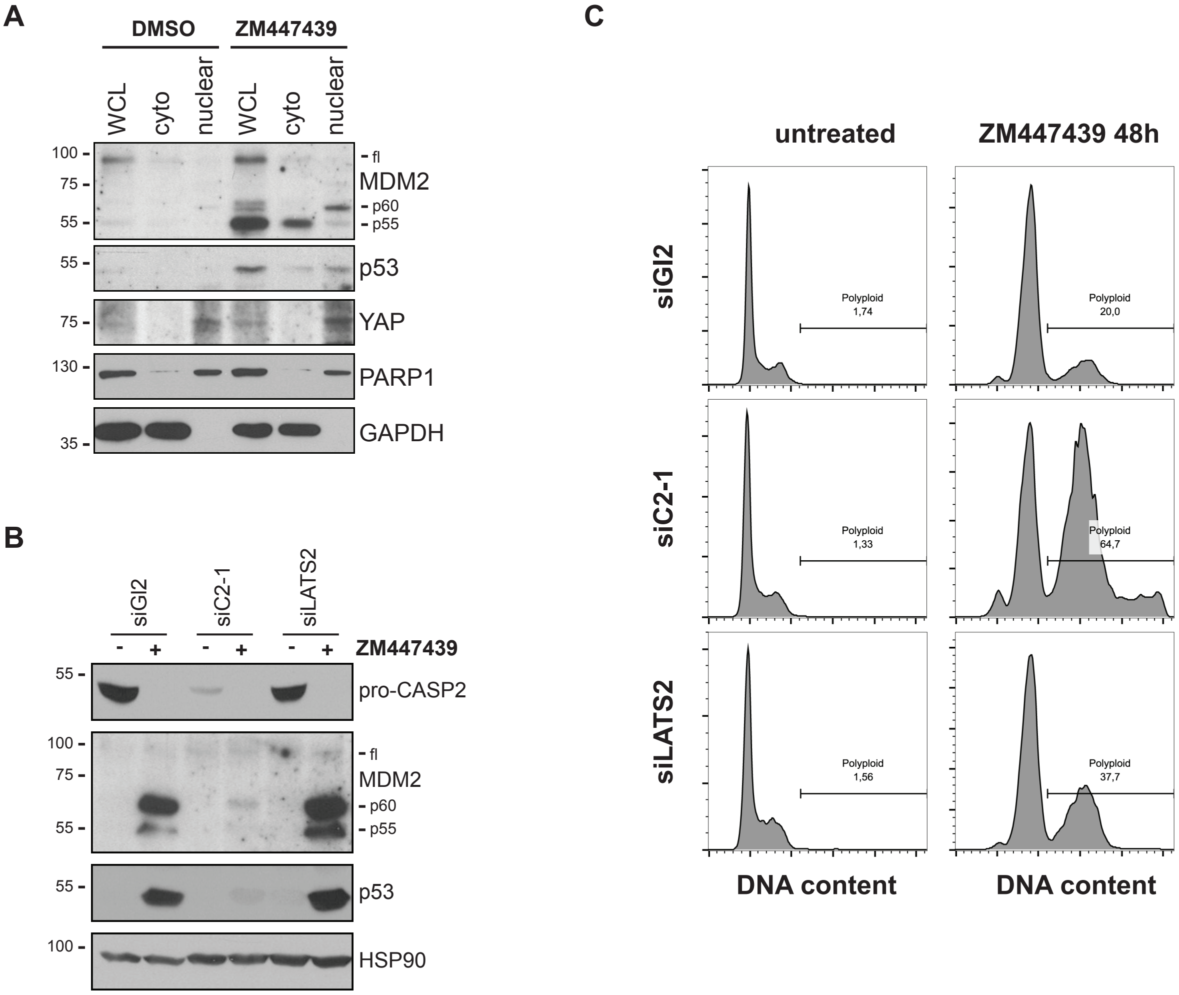
A
DMSO
ZM447439
WCL
cyto
nuclear
WCL
cyto
nuclear
100
75
55
fl
MDM2
p60
p55
55
p53
75
YAP
130
PARP1
35
GAPDH
B
siGl2
siC2-1
siLATS2
-
+
-
+
-
+
ZM447439
55
pro-CASP2
100
75
55
fl
MDM2
p60
p55
55
p53
100
HSP90
C
untreated
ZM447439 48h
siGl2
siC2-1
siLATS2
Polyploid 1,74
Polyploid 20,0
Polyploid 1,33
Polyploid 64,7
Polyploid 1,56
Polyploid 37,7
DNA content
DNA content

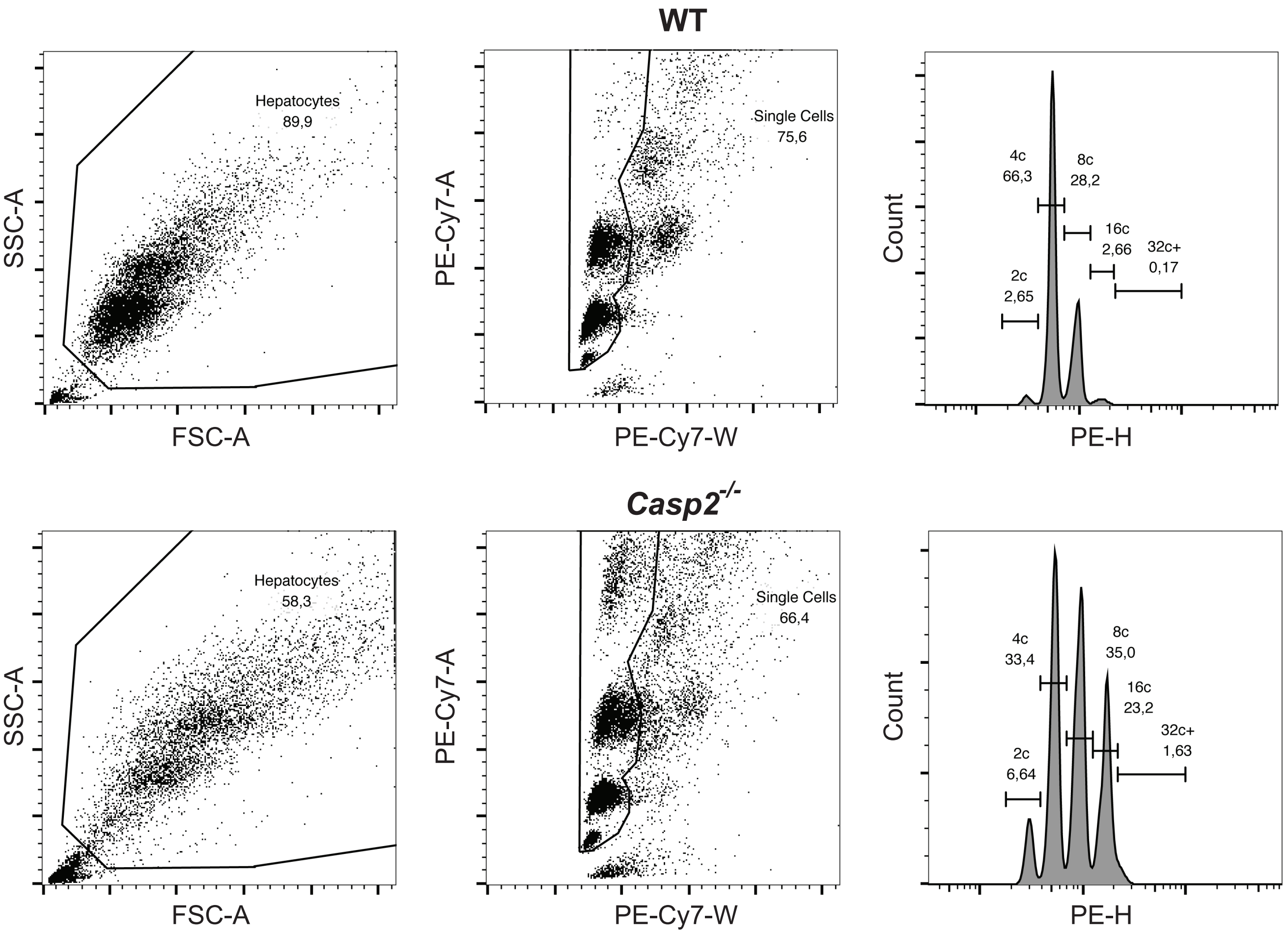
WT
Hepatocytes
89,9
SSC-A
FSC-A
Single Cells
75,6
PE-Cy7-A
PE-Cy7-W
4c
66,3
8c
28,2
16c
2,66
32c+
0,17
2c
2,65
Count
PE-H
Casp2-/-
Hepatocytes
58,3
SSC-A
FSC-A
Single Cells
66,4
PE-Cy7-A
PE-Cy7-W
4c
33,4
8c
35,0
16c
23,2
32c+
1,63
2c
6,64
Count
PE-H

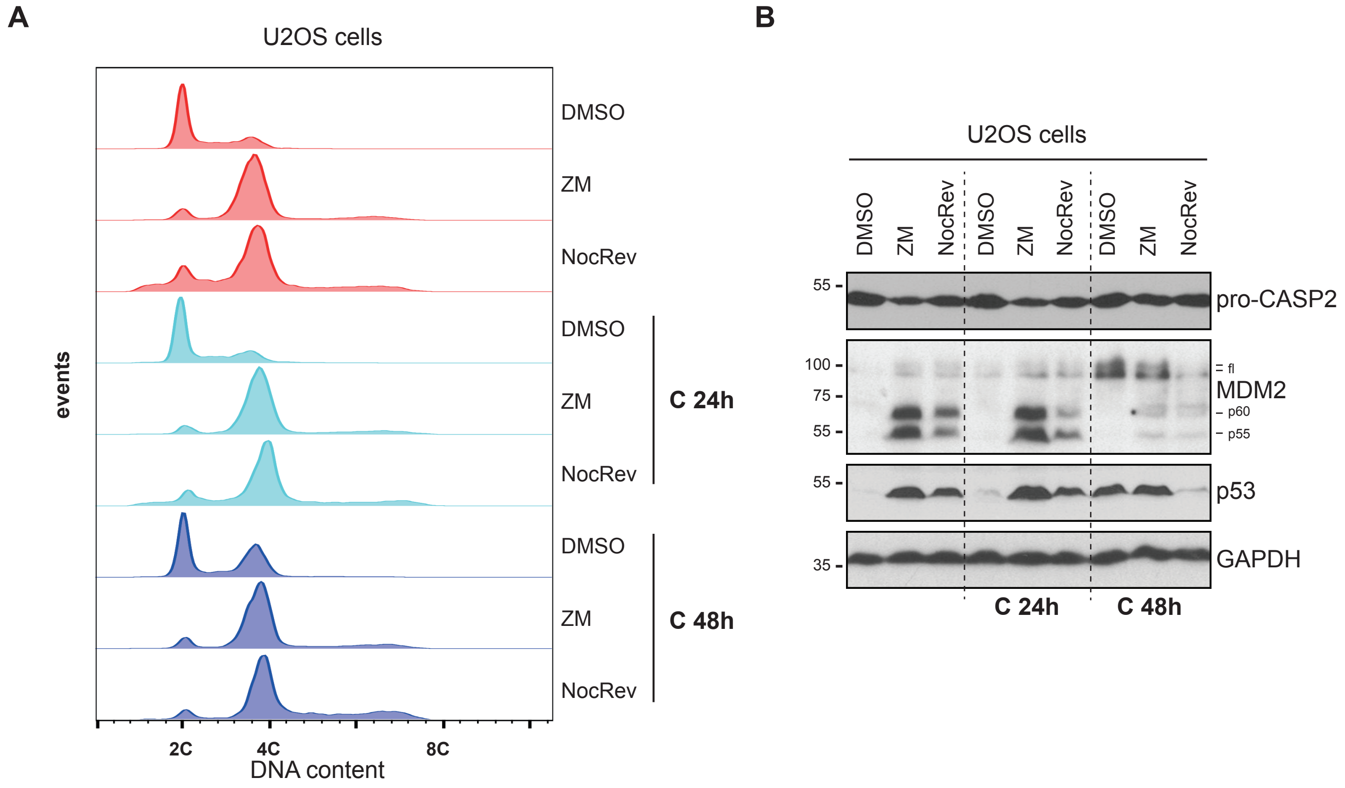
A
U2OS cells
events
DMSO
ZM
NocRev
DMSO
ZM
NocRev
C 24h
DMSO
ZM
NocRev
C 48h
2C
4C
8C
DNA content
B
U2OS cells
DMSO
ZM
NocRev
DMSO
ZM
NocRev
DMSO
ZM
NocRev
55
pro-CASP2
100
75
55
fl
MDM2
p60
p55
55
p53
35
GAPDH
C 24h
C 48h

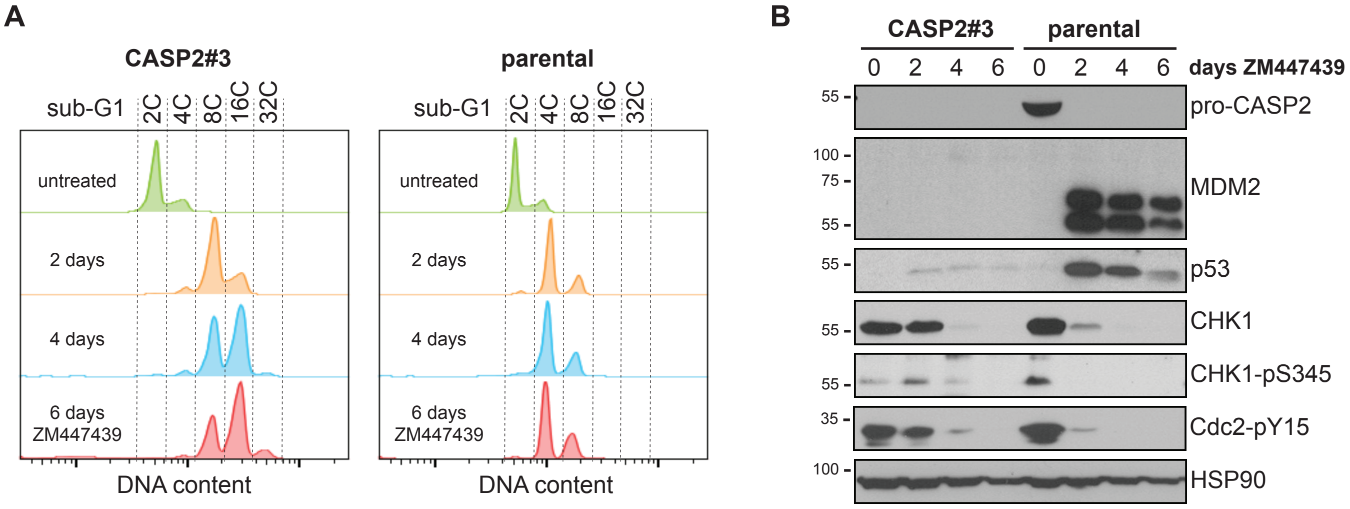
A
CASP2#3
parental
sub-G1
2C
4C
8C
16C
32C
untreated
2 days
4 days
6 days
ZM447439
DNA content
B
CASP2#3
parental
0 2 4 6 0 2 4 6
days ZM447439
55
100
75
35
pro-CASP2
MDM2
p53
CHK1
CHK1-pS345
Cdc2-pY15
HSP90

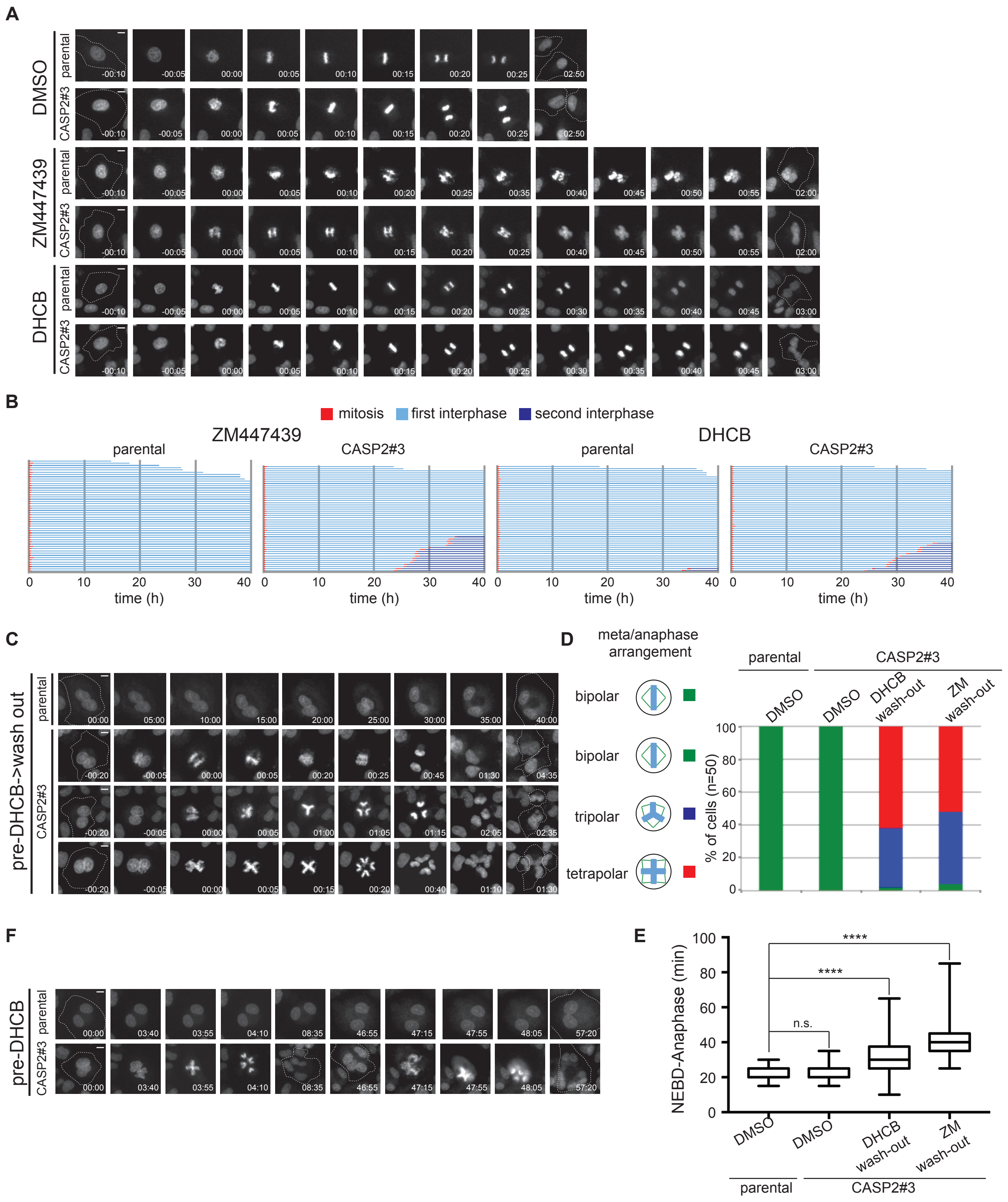
A
DMSO
ZM447439
DHCB
parental
CASP2#3
B
mitosis
first interphase
second interphase
ZM447439
DHCB
parental
CASP2#3
time (h)
C
pre-DHCB->wash out
D
meta/anaphase arrangement
bipolar
bipolar
tripolar
tetrapolar
% of cells (n=50)
DMSO
DHCB wash-out
ZM wash-out
E
NEBD-Anaphase (min)
n.s.
****
F
pre-DHCB

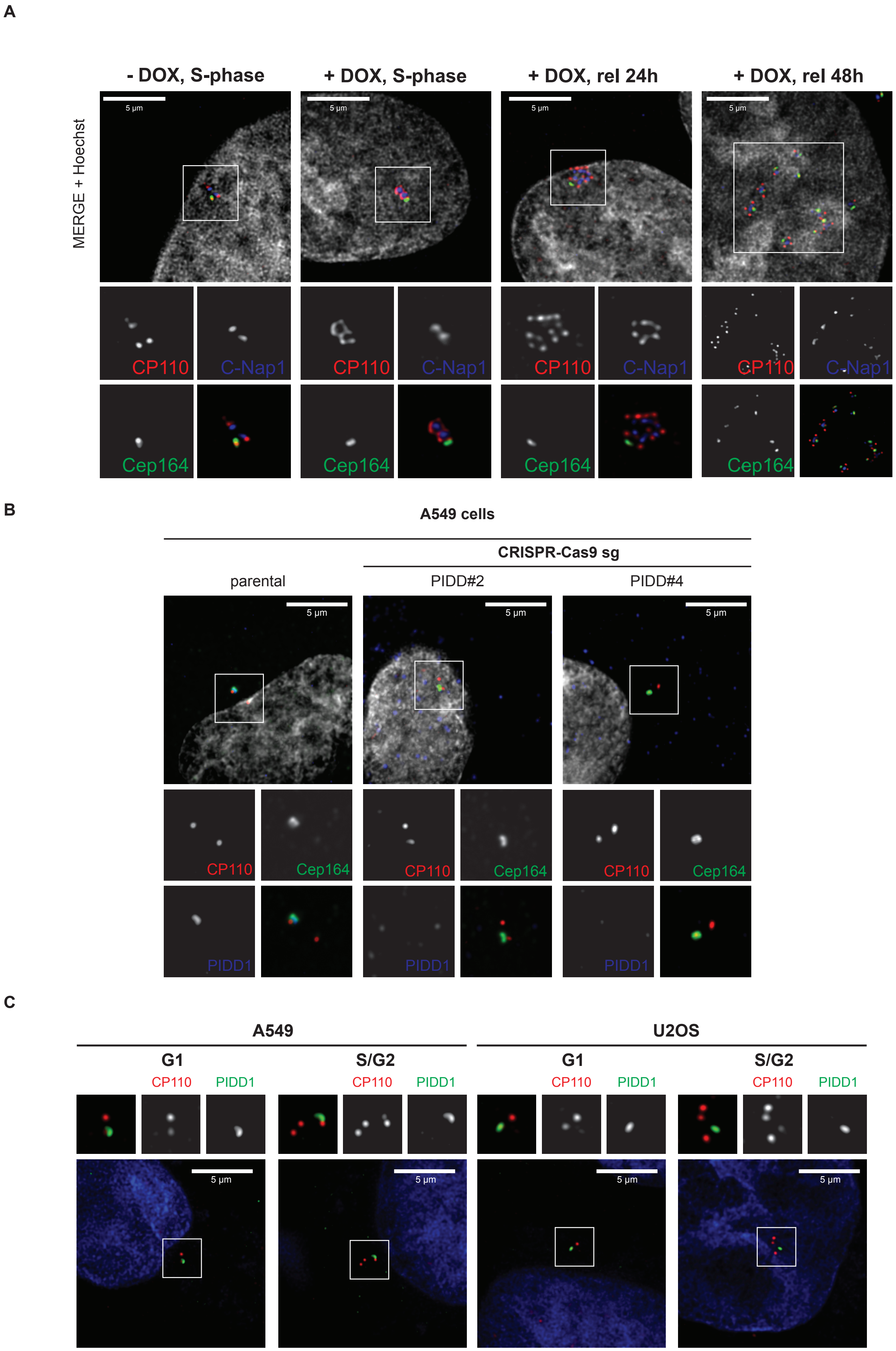
A
- DOX, S-phase
+ DOX, S-phase
+ DOX, rel 24h
+ DOX, rel 48h
MERGE + Hoechst
5 µm
CP110
C-Nap1
Cep164
B
A549 cells
CRISPR-Cas9 sg
parental
PIDD#2
PIDD#4
CP110
Cep164
PIDD1
C
A549
U2OS
G1
S/G2
CP110
PIDD1
5 µm
